# Insights into early continental crust formation from the most ancient heart of Scotland

**DOI:** 10.1038/s41467-026-72076-6

**Published:** 2026-04-21

**Authors:** Silvia Volante, Fernanda Torres-Mancinelli, Jonas Kaempf, Vitor Barrote, Tim Johnson, Christopher Kirkland, Maria Rosa Scicchitano, Lorenzo Tavazzani, Sampriti Basak, Anne-Sophie Bouvier, Annika Dziggel, Axel Gerdes

**Affiliations:** 1 Structural Geology and Tectonics Group, Geological Institute, Department of Earth & Planetary Sciences, ETH Zürich, Zürich, Switzerland; 2 Department of Tectonics and Resources, Institute of Geoscience, Ruhr–Universität Bochum, Bochum, Germany; 3 Curtin Frontier Institute for Geoscience Solutions, School of Earth and Planetary Sciences, Curtin University, Perth, WA Australia; 4 Center for Nuclear Engineering and Sciences, Paul Scherrer Institute, Villigen, Switzerland; 5 GFZ Helmholtz Centre for Geosciences, Potsdam, Germany; 6 Department of Earth and Planetary Sciences, Institute of Geochemistry and Petrology, ETH Zürich, Zürich, Switzerland; 7 Department of Geosciences and Natural Resource Management, University of Copenhagen, Copenhagen, Denmark; 8 Institut des Sciences de la Terre, Université de Lausanne, Quartier UNIL-Mouline, Lausanne, Switzerland; 9 Department of Geosciences, Frankfurt Isotope and Element Research Center (FIERCE), Goethe-University Frankfurt, Frankfurt, Germany

**Keywords:** Precambrian geology, Geochemistry, Petrology, Geology

## Abstract

The paucity of rocks from Earth’s first billion years (4.5–3.5 Ga) limits understanding of early felsic (continental) crust formation and craton development. We present zircon U–Pb, O- and Hf-isotope and whole-rock geochemical data from deformed and metamorphosed (ultra)mafic and felsic rocks of the Archaean Lewisian Gneiss Complex, NW Scotland. The felsic, MgO-rich hornblende-bearing tonalite gneisses contain magmatic zircon populations at c. 3.6, 3.5, and 2.8 Ga, as do enclosed hornblendite pods (>95 vol.% hornblende). Zircons from hbl-tonalite preserve median δ¹^8^O_(zircon)_ of ~5.8‰, indicating limited supracrustal input. The hornblendites have high-MgO (~18 wt%), Cr (up to 5500 µg/g), and Ni (up to 2800 µg/g) concentrations and record slightly higher δ¹^8^O_(zircon)_ median values (~6.3‰). Zircon εHf_(zircon)_ from both lithologies implies a Hadean to early Archaean (c. 4.1–3.9 Ga) depleted mantle source variably modified by low-temperature surface-derived fluids and/or incorporation of Hadean continental crust. We argue that the hornblendites represent vestiges of a hydrated (proto)crust that contributed to the growth of the earliest Archaean continental nuclei.

## Abstract (web summary)

Ancient rocks from northwest Scotland show that early continental crust formed in water-rich environments. The data suggest that primordial water was already present in the lower crust and was recycled from altered surface rocks into deeper crustal levels.

## Introduction

Earth’s Archaean (4.0–2.5-billion-year-old) continental crust is dominated by variably deformed and metamorphosed primitive granitoids of the tonalite–trondhjemite–granodiorite (TTG) suite, most of which were derived from partial melting of hydrated basaltic crust (i.e., amphibolite^1^). However, the geodynamic setting in which this continental crust formed continues to be debated. Whereas some propose an origin in subduction settings^2,3^, others favour partial melting near the base of thick oceanic plateaus in a single-plate (or stagnant-lid) setting^4–6^ driven by mantle plumes^7,8^ or perhaps even bolide impacts^9,10^.

Regardless of the geodynamic setting, it is clear that water played a critical role in TTG genesis^11–16^. The presence of H_2_O lowers the temperature at which basaltic rocks begin to melt and fundamentally influences both the volume and composition of the melt produced. Subduction provides one obvious mechanism for bringing near-surface water to depth^17^. However, density-driven sinking of dense ultramafic–mafic-dominated supracrustal rocks is a plausible alternative during the Archaean, when a hotter mantle may have precluded stable subduction^18,19^. Alternatively, others have demonstrated that meteorite impacts may cause processes akin to transient subduction^20^. Importantly, recent work has shown that hydrated komatiites and other high-MgO ultramafic rocks may have carried significant volumes of mineral-bound water that remained stable to temperatures exceeding 700 °C, permitting fluid-fluxed melting of the basaltic lower crust and/or lithospheric mantle^12,13^. Determining the source and the role of water in the early Earth is fundamental to a better understanding of how ancient continental nuclei formed^14,21,22^.

The origin of Earth’s hydrosphere has long been explained by the delivery of water by comets and asteroids^23^. However, a growing body of evidence suggests that much of the water may have already been present, sequestered within the numerous bodies that accreted to form our planet in the early history of the solar system^24^. Analyses of hydrogen isotopes in olivine-hosted melt inclusions and nominally anhydrous minerals point to a deep mantle reservoir that predates late accretion^25^. These findings suggest that at least part of Earth’s water was already present in the mantle rather than having been solely imported. This interpretation is consistent with the oxygen isotope composition of primitive sodic TTGs derived from source rocks hydrated by primordial (mantle-derived) H_2_O^26^.

Here, we investigate the petrogenetic processes associated with early continental crust formation, including the source of H_2_O, through a case study of the Archaean Lewisian Gneiss Complex (LGC) in NW Scotland. Our approach combines field and petrological observations with major and trace element whole-rock geochemical data, and in-situ U–Pb, O and Hf isotopic and trace element (TE) analyses of magmatic zircon from hornblende-bearing TTG gneisses and enclosed hornblendite pods. We interpret the hornblendites to represent fully hydrated, rigid igneous bodies that were entrained in the TTG melts, either in the magma source region or during magma ascent. Although the rocks have been metamorphosed to granulite-facies conditions during the Neoarchaean^27,28^, they retain key evidence from their earlier Eoarchaean to Hadean history, with implications for crust-forming processes on the early Earth.

## Results

### Sample descriptions and whole-rock geochemistry

**Fig. 1:**
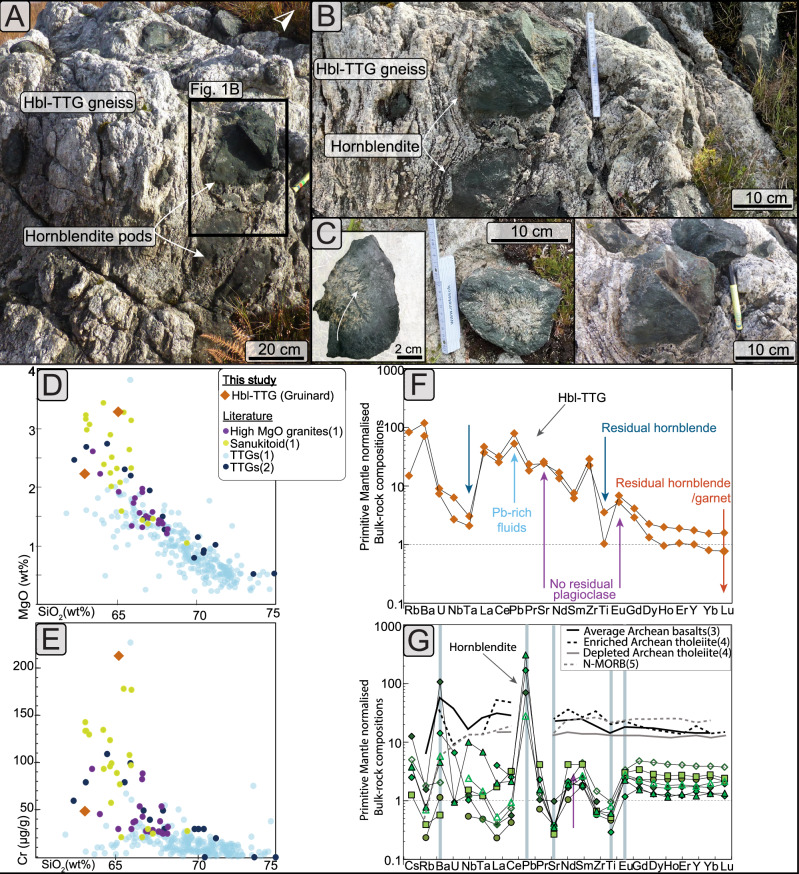
Prominent outcrop-scale features of the hornblendite lenses in hbl- TTGs. **A** Dark green hornblendite pods wrapped by the gneissic foliation in hbl-TTGs. **B** Detail of dark green hornblendite pod with core-rim texture. **C** Dark green hornblendite lenses with a core-rim texture, defined by core with up to 5-cm long randomly oriented amphibole crystals transitioning into finer-grained amphibole-bearing rims. **D**, **E** Compositional variation in MgO and Cr versus SiO_2_ for the investigated hbl-TTG sample. Primitive mantle-normalised trace elements patterns for (**F**) hbl-TTG and (**G**) hornblendite samples investigated in this study. The coloured arrows and the grey bands in correspondence of negative and positive anomalies reflect absence and presence of minerals in the rock. Cited references are: (1)^26^; (2)^92^; (3)^93^; (4)^94^; (5)^95^.

In-situ U–Pb, TE, Lu–Hf and O isotope analyses were performed on zircon from hornblende-bearing tonalite gneisses (hereafter hbl-TTG) and enclosed hornblendite pods (Fig. 1A–C) from the Achnasheen–Gruinard locality in the Gruinard terrane of the LGC^28^ (see Supplementary Information, Supplementary Figs. 1 and 2). At outcrop scale, the hbl-TTG are weakly deformed, coarse-grained, and composed mostly of plagioclase, quartz, and hornblende (Fig. 1A), contrasting with the typical, strongly deformed and finer-grained banded grey gneisses that dominate the LGC^29^. The hornblendite pods are dark green, medium- to coarse-grained rocks and dominated by granoblastic hornblende (≥95 vol.% hornblende), with minor accessory or interstitial phases including quartz, Cr–Ti–Fe oxides and zircon. The hornblendite pods are weakly deformed to undeformed, up to two-metres across and have sharp contacts with the host hbl-TTG gneisses (Fig. 1A). Some hornblendite pods contain cores with prismatic hornblende crystals up to 5 cm long that transition into finer-grained rims up to a centimetre thick (Fig. 1B, C).

The hbl-TTG samples contain plagioclase, quartz, and hornblende with accessory zircon. Secondary biotite replaces hornblende, and apatite and rutile occur within biotite and amphibole–quartz intergrowths that replace hornblende or clinopyroxene (see Supplementary Information, Supplementary Fig. 3). The hbl-TTGs (samples LG2022 and LG2023B) have ~64 wt% SiO_2_ and are sodic (K_2_O/Na_2_O < 0.4), with high Sr/Y ratios (>30), Mg# (49 and 67, where Mg# = 100 x atomic Mg/(Mg + Fe)), MgO (~3.0 wt%), Ni (44 and 168 µg/g) and Cr (48 and 214 µg/g) contents (Fig. 1D–F) relative to most TTGs^26,29^. Although these samples have a sanukitoid-like chemistry, a suite typically characterised by SiO₂ ≤ 60 wt.%, MgO > 6 wt.%, Mg# > 0.60, Cr > 100 µg/g, and Sr and Ba > 500 µg/g, our samples have slightly higher SiO₂ (65 and 63 wt.%), and lower MgO (2.2 and 3.3 wt.%). In primitive-mantle normalised trace element plots, the hbl-TTGs show pronounced negative anomalies for Nb, Ta and Ti, and positive anomalies for Pb, Zr, Eu and Sr (Fig. 1F).

The associated hornblendite pods (Supplementary Data 1) commonly exhibit large (~4 mm) granoblastic amphibole crystals with diffuse to patchy zoning, mantled by sub-mm-thick pale green acicular to prismatic actinolite rims. Fine-grained amphibole–quartz intergrowths mimic domains that may represent pseudomorphs after primary magmatic clinopyroxene, although no relics were observed. When a core–rim structure at the pod-scale is present, cores contain randomly oriented, prismatic amphibole crystals that are partly replaced by acicular actinolite aggregates. Towards the rims, grain size decreases. Quartz, when present, occurs as thin films along amphibole grain boundaries or as small veinlets. Cr–Ti–Fe oxides are widespread, most enclosed in amphibole cores but also associated with actinolite rims. Rare subhedral zircon grains up to ∼400 µm in size are enclosed in large amphibole crystals. These pods are characterised by 49–53 wt% SiO_2_, 16–19 wt% MgO, 6–12 wt% CaO, and low TiO_2_ (<0.2 wt%) with Mg# of 68–75. Hornblendite pods with texturally distinct core and rim structures (Fig. 1B, C) have FeO contents decreasing from core (13–16 wt%) to rim (11 wt%), increasing CaO (from 6–9 wt% to 11 wt%), and decreasing Ni contents (from >2000 to 500 µg/g). They exhibit Cr contents up to 5620 µg/g, Ni up to 2850 µg/g, and V between 20 and 105 µg/g. Elemental maps reveal Cr and Ti zoning in amphibole crystals with concentrations decreasing from core to rim (see Supplementary Information, Supplementary Fig. 3). These elements are also concentrated in Cr–Ti–Fe oxides (chromite), which occur as inclusions in amphibole cores. Primitive mantle-normalised TE patterns for the whole rock composition of the hornblendites reveal depletion in Cs, Rb, U, Ti, La, and Sr, mild enrichment in Ba, and flat middle to heavy rare earth element (M- to H-REE) patterns, with positive anomalies in Ba, Pb, Nd, Sm and Eu (Fig. 1G).

### Zircon U–Pb geochronology and geochemistry

**Fig. 2:**
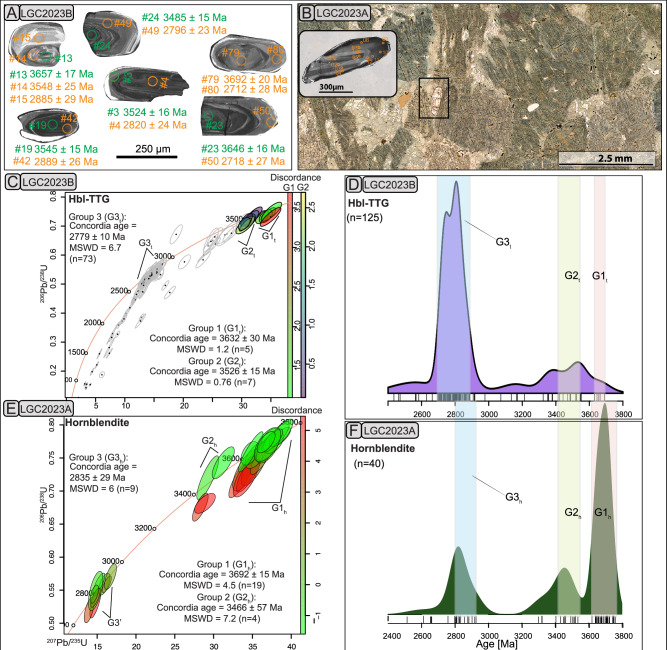
Geochronology results for zircon. **A**, **B** Representative cathodoluminescence (CL) images of analysed grains with the spot location (green and orange for analysis from Curtin University and ETH, respectively). **C**–**F** Concordia diagrams and kernel density estimation plots of ^207^Pb/^206^Pb ages ± 2SE (95% confidence) of zircon analyses from hbl-TTGs and hornblendite, respectively.

**Fig. 3:**
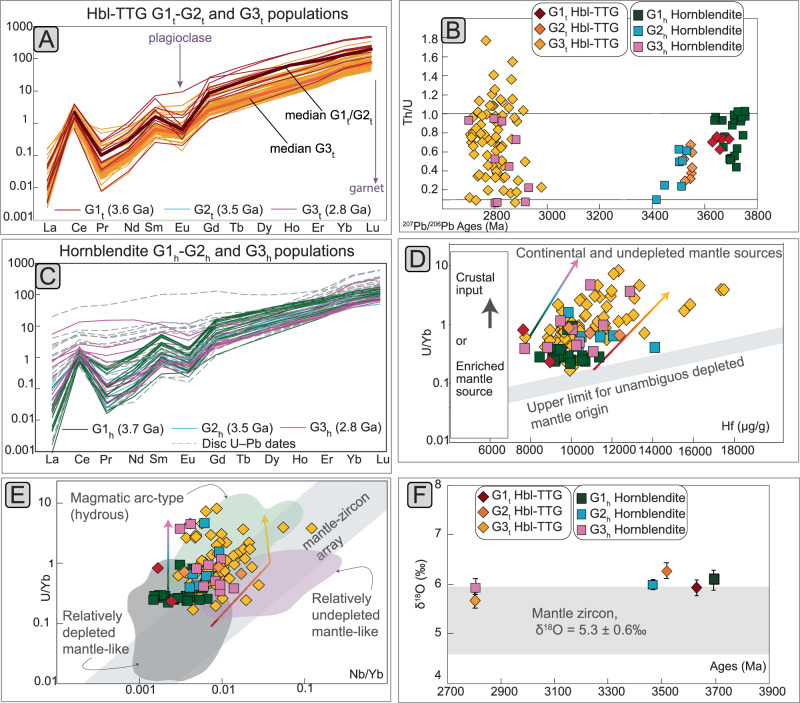
Trace-element and O-isotope compositions of zircon from hornblendite and hbl-TTGs. Primitive mantle-normalised rare earth element (REE) patterns for (**A**) the older and younger Hbl-TTG components; **B** Th/U ratio versus ages (Ma); **C** REE patterns for the hornblendite populations; **D** U/Yb versus Hf and **E** U/Yb versus Nb/Yb discrimination diagrams^31^; **F** Median O-isotope compositions of zircon and associated 2 SD versus ages (Ma) for hbl-TTG and hornblendite samples from the Lewisian Gneiss Complex.

The hbl-TTG contains magmatic zircon cores up to c. 3700 Ma old, with 20 concordant (<5% discordance) ^207^Pb/^206^Pb dates older than 3300 Ma (see Supplementary Information, Supplementary Figs. 2 and 4). These core domains display oscillatory zoning typical of magmatic zircon, and are partially dissolved and characterised by overgrowths that also exhibit oscillatory zoning (Fig. 2A). Three zircon populations are identified based on internal textures, Th/U ratios, and relative TE content (Figs. 2C, D and 3A–E). Group 1 in the TTGs (G1_t_) represents the oldest zircon population with five analyses that define a concordia age of 3632 ± 30 Ma (MSWD = 1.2, *n* = 5; all age uncertainties are presented at 95% confidence—2SE) and Th/U ratios between 0.62 and 0.76 (median = 0.7; Fig. 3B). Group 2 (G2_t_) zircon analyses are either rims on c. 3600 Ma cores or concentrically zoned grains. This population comprises seven analyses that yield a concordia age of 3526 ± 15 Ma (MSWD = 0.76, *n* = 7) with Th/U ratios between 0.30 and 0.66 (median =  0.46; Fig. 3B). Group 3 (G3_t_) is the dominant population and defines an over-dispersed concordia age of 2779 ± 10 Ma (MSWD = 6.7, *n* = 73), where high MSWD indicates some radiogenic-Pb remobilisation. Also, recent Pb-loss events contributing to discordance towards c. 500 Ma are observed (Fig. 2C). Cathodoluminescence images indicate that eight analyses with ^207^Pb/^206^Pb dates ranging between 3485 ± 26 Ma and 3351 ± 27 Ma represent core–rim mixtures. Overall, G1_t_ and G2_t_ zircon grains/domains are slightly more enriched in HREE with G1_t_ showing a more pronounced negative Eu anomalies (Eu/Eu* = Eu/(Sm*Gd)^0.5^, with Eu/Eu* = 0.28–0.70) than G2_t_ (Eu/Eu* = 0.04–0.4) and G3_t_ (Eu/Eu* = 0.1–0.6; Fig. 3A).

Zircon grains within the hornblendite are sparse but are commonly found within the core domains of the hornblendite pods. These zircon grains display a diverse range of morphologies, including rounded (e.g., zrn1) and elongated prismatic crystals (e.g., zrn3) that are up to ~700 µm long and have complex internal zonation with discontinuous rims (Fig. 2B, Supplementary Information, Supplementary Fig. 5). Three zircon populations are identified based on internal textures, Th/U ratios, and relative TE content (Figs. 2E, F and 3A–E). Group 1 in the hornblendite (G1_h_) represents the oldest zircon population with nineteen analyses that define a concordia age of 3692 ± 15 Ma (MSWD = 4.5, *n* = 19) and Th/U ratios between 0.5 and 1.0 (median = 0.8). Group 2 zircon in the hornblendite (G2_h_) comprises four analyses which yield a concordia age of 3466 ± 57 Ma (MSWD = 7.2, *n* = 4) with Th/U ratios between 0.23 and 0.5 (median = 0.4). Zircon rims at c. 3500 Ma (G2_h_) are consistent with the G2_t_ population for the hbl-TTGs. Only one ^207^Pb/^206^Pb date of 3419 ± 40 Ma was recovered for the outermost bright rim of a zircon grain with distinctively lower Th/U ratio of 0.09. Finally, Group 3 zircon in the hornblendite (G3_h_) from CL-bright zircon cores defines a concordia age of 2835 ± 29 Ma (MSWD = 6, *n* = 9), with Th/U ratios between 0.1 and 0.7 (median = 0.1). The high MSWDs are consistent with over-dispersed data, which may indicate more than one episode of radiogenic-Pb loss. Concordant grains forming G1_h_–G3_h_ populations exhibit distinct negative Eu anomalies (Fig. 3C).

In addition to REE patterns, plots of Hf versus U/Yb and Nb/Yb versus U/Yb and their covariation, provide further information about zircon crystallisation environments and sources^30,31^. For both hbl-TTGs (G1_t_, G2_t_, and G3_t_) and hornblendite (G1_h_, G2_h_, and G3_h_) zircon populations, a progressive enrichment in Hf and U relative to Yb gives higher Hf concentrations and an increase in the U/Yb ratio of the younger zircon grains (Fig. 3D). The Nb/Yb vs U/Yb diagram shows that, while G1_h_ plots on the mantle-zircon array, G2_h_ and G3_h_ define a vertical trend in the field of hydrous magmatic arc rocks. For the hbl-TTGs, G1_t_ also falls within this field, whereas both G2_t_ and G3_t_ plot towards the upper limit of the mantle array towards the hydrous magmatic arc field (Fig. 3E).

### Oxygen and hafnium isotopes in zircon

Zircon from the hbl-TTGs displays subtle variations in δ^18^O_(zircon)_ across age groups (see Supplementary Information, Supplementary Fig. 6). A total of twelve analyses for G1_t_ (c. 3630 Ma) and G3_t_ (c. 2800 Ma) zircon, cluster within or at the upper limit of mantle values (δ^18^O = 5.3 ± 0.6‰—2 SD)^32^, with median values of 5.94 ± 0.2‰ and 5.69 ± 0.3‰, respectively (Fig. 3F). Group 2 (G2_t_) zircons (c. 3520 Ma) yield an elevated median of 6.23 ± 0.2‰ (three analyses), requiring input from a heavier oxygen component. The hornblendite zircon grains of c. 3690 Ma yield a δ^18^O_(zircon)_ median of 6.1 ± 0.2‰. For G2_h_ and G3_h_ only one robust data point for each yield δ^18^O_(zircon)_ of 5.8 ± 0.1‰ and 5.93 ± 0.2‰, respectively.

**Fig. 4:**
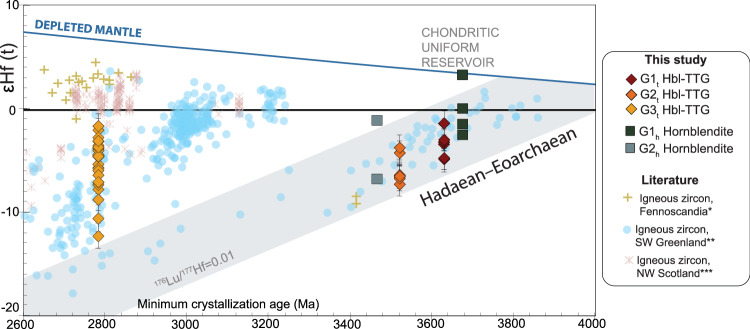
Zircon εHf isotope evolution diagram of Eo- and Mesoarchaean zircon from Hbl-TTG and hornblendite samples from the LGC. The U–Pb ages used are estimated minimum crystallisation ages for each TTG sample. Note major addition of juvenile material at c. 2800–3000 Ma during the formation of the Archaean crust from the Lewisian Gneiss Complex and partial contribution from the c. 3700 Ma, Eoarchaean crust from Greenland. Depleted mantle values: ^176^Lu/^177^Hf = 0.0388, ^176^Hf/^177^Hf = 0.28325^96^. CHUR values: ^176^Lu/^177^Hf = 0.0336, ^176^Hf/^177^Hf = 0.282785^89^. λ176Lu = 1.867 × 10 − 11 a^−1^ ^97^. Error bars for Hf data are shown at two standard errors. Source data information for this figure is provided in Supplementary Data 6. Published dataset are light blue circles for TTG samples from Greenland, also part of North Atlantic Craton^52,54^, yellow crosses for TTG samples from Fennoscandia^53^, pink stars reflect TTG data from NW Scotland^34^, and the grey shaded field reflects crustal evolution of Greenland^54^.

The εHf in zircon from the hbl-TTG samples, recalculated to the time of crystallisation, reveals distinct source characteristics across age groups. Group 1 zircon (G1_t_, c. 3630 Ma) yields subchondritic εHf_(zircon)_ values between –5.0 and –1.5, whereas G2_t_ (c. 3520 Ma) is on average more evolved, ranging from –7.4 to –3.1. The younger G3_t_ population (c. 2800 Ma) exhibits more significant negative εHf_(zircon)_ values (–13 to –2.2; Fig. 4). Assuming a ^176^Lu/^177^Hf of 0.12^33^, G1_t_ and G2_t_ have Hadean median model (mantle extraction) ages of c. 4100 Ma. By contrast, G3_t_ has a Paleoarchaean median model age of c. 3460 Ma. In the hornblendite sample, εHf_(zircon)_ values range from –7.1 to +3.1, consistent with a more juvenile source at c. 3690 Ma. The median model age of c. 3900 to 4000 Ma is consistent with derivation from an Eoarchaean to Hadean mantle source. Due to spatial resolution limitation of employed in situ technique, no analyses were possible on the G3_h_ domains, and only two data points were acquired on the G2_h_ domains.

## Discussion

### Petrogenesis of Eoarchaean high-MgO hornblendite and hbl-TTG

There is abundant zircon U–Pb data from the LGC^28,34^, from which the previous oldest TTG is dated at c. 3135 ± 5 Ma^35^. In contrast, previous ^207^Pb–^206^Pb and ^147^Sm–^143^Nd isotopic data for mineral and whole-rock separates from mafic rocks in the LGC, yielded ages of c. 3300 Ma interpreted as the source of associated tonalitic melts^36^. Here, we report zircon ages from hornblende-bearing high-MgO TTGs and enclosed hornblendite pods that are older than 3600 Ma, providing direct evidence for the existence of evolved Eoarchaean crust in NW Scotland. The identification of a crustal component half-a-billion years older than previously recognised highlights the important role of high-MgO ultramafic–mafic rocks in the generation of primitive continental (TTG) crust and provides a temporal link between the LGC and the rest of the North Atlantic Craton. Zircon populations from the hbl-TTG yield ages of c. 3630 Ma (G1_t_), c. 3520 Ma (G2_t_), and c. 2800 Ma (G3_t_). A positive correlation between G1_t_ and G2_t_ and Th/U ratios indicates that older grains generally have higher Th/U values. G3_t _reports a wide range in Th/U > 0.1, likely indicating hydrothermal and magmatic zircon growth during crust formation. Texturally, G1_t_ and G2_t_ zircons appear as oscillatory zoned cores partially overgrown by G3_t_ rims, consistent with inherited relicts of eroded or buried crustal material, indicating the presence of nearby Eoarchaean crust^37^. Combined, these observations further support the magmatic origin for G1_t_ and G2_t_, although a metamorphic origin for G2_t_ cannot be excluded. Distinct εHf_(zircon)_ evolution trends for G1_t_ /G2_t_ versus G3_t_, together with a juvenile component at c. 2800 Ma, indicate that the older zircon populations G1_t_ /G2_t_ are best explained as inherited from nearby older crust, which was subsequently recycled (Fig. 4).

Although ultramafic–mafic rocks are typically zircon-poor due to low Zr content, hornblendite pods in the LGC contain large zircon crystals (up to ~700 µm). These yield ages of c. 3690 Ma (G1_h_), c. 3470 Ma (G2_h_), and c. 2840 Ma (G3_h_). Zoned cores from the older grains evidence resorption textures and younger rims and the Th/U values decrease from 0.9 to 0.7 in the older grains to ~0.1 in the youngest c. 2840 Ma zircon population (G3_h_). Rims with ^207^Pb/^206^Pb dates of ca. 3470 Ma likely formed during the same event recorded by G2_t_ hbl-TTG zircon population. The zoning, TE budget and size of the ca. 3690 Ma zircon grains (G1_h_) implies crystallisation in a hot, water-rich environment^38^ (Fig. 3), whereas the patchy zoning from rim to core of G2_h_ may support a hydrothermal/metamorphic origin for this age population (see Supplementary Information, Supplementary Fig. 5). The younger cores and rims (c. 2840 Ma) align with the major crust-forming event in the LGC^28,39^.

Hydrated ultramafic rocks, including komatiites, have been invoked as key sources of H_2_O, with this fluid released at relatively high temperatures (700 − 750 °C) during burial, irrespective of the burial mechanism^13,15^. In the LGC, ultramafic rocks have previously been interpreted as either cumulates from early TTG magma formation^40,41^, or as primary layered intrusions derived from partial melting of a hydrous mantle^42^. Sharp and irregular contacts between the hornblendite pods and the hbl-TTG host indicate that the ultramafic rocks were incorporated as rigid bodies into the tonalite magma suite. The hornblendite pods display high whole-rock MgO, Cr, and Ni contents and are monomineralic, with core-to-rim FeO, Cr and Ni depletion and Ca enrichment in individual amphibole grains. These textural and chemical features are consistent with a mantle-derived ultramafic rock that was fully hydrated in the source region of the TTGs. These rocks interacted with hydrous melt during entrainment and ascent. An entrainment in the Eoarchaean source is also supported by the strong overlap of zircon ages from the different age groups in hornblendite and hbl-TTG (Fig. 2D, F). The hornblendites thus may represent residual hydrated mafic crust or hydrous mantle-derived cumulate entrained during fluid-fluxed partial melting of mafic crust at 700–750 °C^12,43^. Amphibole textures and zircon morphologies—such as randomly oriented, cm-sized amphibole grains and prismatic Eoarchaean zircon associated with hornblende—coupled with O- and Hf-isotopes support zircon growth in a water-rich environment (Figs. 1C, 2B).

The hbl-TTGs reflect the most primitive TTG magmas in the LGC and host the oldest known zircon components from Scotland. Their high MgO, Cr and Ni contents suggest derivation from partial melting of a high-MgO, hydrous mafic source. Negative Nb, Ta and Ti and positive Zr anomalies along with HREE depletion reflect retention of amphibole and/or garnet in the source^44,45^, whereas positive Pb anomalies are consistent with H_2_O-fluxed melting^46^, potentially involving mantle-derived fluids released by subduction or sinking of ultramafic–mafic crust into the lowermost crust or lithospheric mantle at 700–750 °C^13,14,17^.

### Eoarchaean primitive melt and fluid sources

The δ^18^O_(zircon)_ values from the high-MgO hbl-TTGs at c. 3630 Ma (G1_t_: 5.94 ± 0.2‰), c. 3520 Ma (G2_t_: 6.23 ± 0.2‰), and c. 2800 Ma (G3_t_: 5.69 ± 0.3‰) fall within or just above mantle values (Fig. 3F), consistent with values observed in most other TTGs^34,47,48^. These data suggest interaction between primitive magmas and a mantle-derived fluid source, with subordinate contribution from a more evolved fluid component. This is further supported by the Hf and Nb/Yb versus U/Yb discrimination diagrams, which suggest a dominant mantle source for G1_t_, G2_t_, and G3_t_ with a subordinate crustal input for the latter straddling towards higher U/Yb ratios (Fig. 3D, E). Zircon grains from a hbl-TTG plotting within the mantle array, likely crystallised from melts derived either from mafic protoliths or from TTG crust modified through prolonged interaction with mafic magmas. In contrast, zircon samples within the magmatic arc-type field were interpreted as derived from anatexis of the volumetrically dominant TTG^49,50^.

Similarly, hornblendite zircon at c. 3690 Ma straddles the upper limit of the δ^18^O_(zircon)_ mantle range (median of 6.4 ± 0.2‰) (Fig. 3F), implying interaction with mantle-derived fluids and potentially a minor supracrustal contribution. Although only one data point was recovered for G2_h_ and G3_h_, this data is consistent with corresponding populations of the hosting hbl-TTG. This slightly higher isotopic signature for G1_h_ may result from interaction of the (ultra)mafic protoliths with an older crustal component at depth or from high-MgO basalts that experienced near-surface hydrothermal alteration before sinking into the lower crust. The release of ^18^O-enriched fluids could have hybridised the lithospheric mantle, contributing to TTG source rocks^5,13,14,26^. Nonetheless, a dominant mantle-derived source for the hornblendite is supported also by the Hf and Nb/Yb versus U/Yb variation diagram, suggesting that G1_h_, G2_h_ and G3_h_ zircon populations grew from magmas derived from a hydrous mafic source, with subordinate crustal interaction (Fig. 3D, E). We suggest that the hornblendite pods enclosed in the primitive hbl-TTGs from the LGC may represent fragments of an early mafic–ultramafic protocrust.

Hafnium isotope compositions of magmatic Eoarchaean zircon from the North Atlantic Craton^51–53^ indicate derivation from Hadean mantle sources^54^. The calculated mantle extraction ages (c. 4100 Ma for Groups 1 and 2 for the hbl-TTG and c. 4000–3900 Ma for G1_h_ for the hornblendite) indicate that the hbl-TTG magmas may have been derived from a Hadean protocrust, whereas the c. 3690 Ma hornblendite represents a younger juvenile influx within the Hadean–Eoarchaean crustal evolution trend^52,54^. The presence of zircon overgrowths at c. 3500 Ma on both hornblendite and hbl-TTG zircon, indicates a shared metamorphic/hydrothermal or magmatic event, implying proximity of these source domains by that time. By c. 2800 Ma, εHf_(zircon)_ values in the LGC become more negative (∼ –13), coeval with extensive crustal growth and similar trends in Greenland (Fig. 4). Although a minor Pb-loss component could have affected the wide εHf_(zircon)_ range across the zircon populations and, particularly, at 2800 Ma, the evolved (negative) εHf_(zircon)_ signature reflects to a greater extent mixing between a juvenile, MgO-rich mantle-derived source and residual felsic Eoarchaean to Hadean crust (see Supplementary Information, Supplementary Fig. 7). Between 3200 and 2800 Ma, both NW Scotland and Fennoscandia record a change in magma sources, marking a widespread mantle input event^54^. This isotopic rejuvenation has been identified as a global signature that diluted the primordial Hadean-to-Eoarchaean crustal evolution trend during the Mesoarchaean, coinciding with the inferred peak in mantle potential temperature^55^. Collectively, these findings support TTG formation from hydrous mafic sources derived from a mantle domain with limited crustal reworking.

### Tracing the role of fluids in the early Earth

There is broad agreement that many detrital and magmatic zircon grains from the Hadean and Eoarchaean—such as those from the Jack Hills, Australia^56^, the Barberton and Nondweni greenstone belts, South Africa^57^, the Isua and Akilia terranes, Greenland^58^, and the Acasta Gneiss Complex, Canada^59^—preserve chemical and isotopic signatures indicative of crustal recycling. These ancient zircon crystals offer a unique archive of early Earth processes, suggesting that water-rich fluids played a central role in the formation and reworking of the earliest continental crust. The average nature of these fluids appears to have evolved over time, with primary magmatic zircon oxygen isotope compositions in Archaean cratons showing a fundamental shift between c. 3000 and 2500 Ma, as δ^18^O values rose from mantle-like (~5.3‰) to more enriched values (>8‰). This shift in δ^18^O values reflects increasingly altered or recycled components in magmatic sources^9,26,60^. Although δ^18^O signatures were modulated by diachronous tectonic, magmatic, and metamorphic processes, early Archaean zircons typically record interaction with dominantly lighter, fluid-derived oxygen reservoirs, consistent with pervasive hydration in early crustal environments^59^.

Mantle and sub-mantle oxygen isotope signatures of the oldest magmatic zircon grains from Archaean cratons are interpreted as indicative of partial melting at shallow crustal levels of a Fe-rich and hydrothermally altered crust, characterised by low Sr/Y and Gd/Yb ratios^9,61^. Importantly, sub-mantle oxygen isotope values for Eoarchaean to Hadean zircon indicate partial melting of near-surface altered crust that interacted with meteoric- or seawater^14,16^. The origin of shallow partial melting has been associated with meteorite impacts during the early stages of Earth evolution^10,62,63^, while others have suggested an origin within a tectonic environment comparable to modern Iceland^61^. While δ^18^O values higher than >9‰ are typically attributed to post-magmatic alteration^64^, early (>3700 Ma) magmatic zircon with oxygen isotopic signatures that straddle towards the upper limit (i.e., heavy) of mantle values is interpreted as evidence for the interaction between surface water and magmas^56^. These isotopic signatures were reported for high-MgO primitive tonalite with high Sr/Y ratios^26^, consistent with partial melting of a high-MgO basaltic source at mid-to lower crustal levels (30–40 km), in the presence or absence of a subordinate, more evolved and isotopically fractionated supracrustal component that exchanged oxygen isotopes with the hydrosphere at low temperatures.

Recent studies have demonstrated that high-MgO mafic and ultramafic rocks (e.g. altered greenstones) can retain large quantities of crystal-bound water up to temperatures exceeding 700 °C^13,15,65^. Dehydration of these water-rich ultramafic–mafic rocks occurred in the early Archaean in the lower crust or lithospheric mantle, triggering fluid-fluxed melting of the overlying crust or directly providing the hydrous basaltic source, contributing to the formation of high-MgO and Sr/Y ratios primitive tonalitic magmas. These petrogenetic processes are considered to account for the slightly enriched δ^18^O values recorded by the hornblendite and MgO-rich hbl-TTG, indicating that high-MgO ultramafic–mafic pods within primitive hbl-TTGs, even though limited within the Archaean rock record, can retain key information about the processes responsible for crustal growth in the early Earth. The supra-mantle δ^18^O values in hornblendite suggests that water was not only present but was carried from the Earth’s surface to deeper crustal levels, where it was exsolved and assimilated during the interaction with the overlying lower crust or lithospheric mantle^13,14^. The interplay between hydrated ultramafic rocks and sodic, high-MgO tonalitic magmas highlights a potential mechanism for water storage and recycling of water-rich components in the early Earth, providing insights into the importance of hybrid fluid sources in crustal growth processes and mantle interaction during the formation of the most ancient continental nuclei.

This work highlights that (i) water-saturated hornblendite pods enclosed in primitive hbl-TTGs reflect the presence of a water-rich environment on the early Earth, which was key for the formation of primitive Archaean crustal nuclei; (ii) some primordial water was already present within the hydrous lower crust; (iii) near-surface, altered and hydrous ultramafic–mafic crust may have transported water with a supracrustal isotopic signature to depth, delivered during the recycling of upper crustal material to lower crustal levels.

## Methods

### Micro-X-Ray fluorescence elemental mapping

Six samples (LG2023A, LG2023C, LGC2024-4B, LGC2024-4A, LGC2024-2, LGC2024-1B and LGC2024-1A; Supplementary Data 1) were mapped for a suite of elements (including Al, Ca, Cr, Fe, K, Mg, Mn, Na, Ni, P, Si, Ti, and Zr) by micro-X-Ray-Fluorescence (XRF). The elemental maps provided an overview to better visualise element distribution within amphibole grains (see Supplementary Information, Supplementary Fig. 3).

A Bruker M4 Tornado Plus micro-XRF instrument (Bruker Nano GmbH, Berlin) was used at the Department of Geosciences and Natural Resource Management, Copenhagen of University, Denmark. The instrument is a non-destructive analyser that enables fast determination of elements and their distribution on a flat sample surface^66^. The micro-XRF instrument uses a Rh X-ray source with a polycapillary lens that focuses the X-rays in combination with a collimator for reducing the aperture. The signal is captured with two XFlash® silicon drift detector (130 eV resolution). The beam size can go down to 20 μm and elements from Na to U can be detected. The micro-XRF instrument detects individual elements by fluorescence X-rays (secondary X-rays) emitted when the sample is bombarded with high energy X-rays. From Moseley’s law the peak of the lines increases approximately at the square root of the atomic number from the emitted element, so e.g. Fe has a higher intensity than Si^67^. The maximum depth of recorded fluorescence X-rays, the XRF saturation depth, varies with atomic number^68^. Minerals with heavier elements thus have a deeper XRF saturation depth than minerals with lighter elements. The XRF signals attenuate by depth and the XRF from Si has lower energy and is attenuated by the surroundingm^66^. The measured samples were placed in the sample chamber under low-vacuum conditions (2 mbar) to avoid Ar absorption and to facilitate the detection of light elements. The Rh X-ray tube energy was set at 50 kV and a current of 600 µA. Measurements were performed using a 20 μm step size, 20 μm beam size, and an acquisition time of 20 ms per pixel. Spectral quantification was performed in standardless mode using M4 Tornado software.

### Zircon mount preparation

Zircon grains were separated from selected samples (Supplementary Data 1) at the mineral separation facility at the Ruhr-University Bochum by standard jaw crushing, milling, and Wilfley table to concentrate the heavy mineral constituents. Zircon fractions were separated using a Frantz isodynamic separator for magnetic separation and lithium heteropolytungstate (LST) heavy liquids. Zircon grains, together with zircon reference materials, were mounted in a one-inch epoxy disk and polished to reveal the approximate centre of the zircon grains. For the hornblendite, zircon grains from thin section samples were analysed only for U–Pb geochronology. Whereas zircon crystals from the hornblendite samples that were analysed for both U–Pb geochronology and oxygen isotopes were drilled out from the thin sections using a 2-mm diameter microdrill. The drilled sample material was subsequently mounted together with the reference materials in a one-inch resin epoxy disk for oxygen isotope analysis at GFZ and in a one-inch indium mount for oxygen isotope analysis at the SwissSIMS laboratory (University of Lausanne). The mounts were carbon-coated to perform backscattered electron (BSE) and cathodoluminescence (CL; see Supplementary Information, Supplementary Figs. 4 and 5) imaging using a Field Emission-Scanning Electron Microscope of the Zeiss Ultra Plus at the GFZ Helmholtz Centre for Geosciences in Potsdam and a TFS Quanta 200 F SEM at ScopeM ETH Zürich, respectively, to verify the spot positioning and provide information on internal textures to aid further interpretation.

### Whole-rock major and trace-element analyses

Whole-rock major and trace elements were determined at Bureau Veritas, Perth, Western Australia (BV in Supplementary Data 2). Major and minor elements (Si, Ti, Al, Cr, Fe, Mn, Mg, Ca, Sr, Ba, Na, K and P) were determined by X-ray fluorescence (XRF) spectrometry on a fused glass disk and loss on ignition (LOI) was determined by robotic thermogravimetric system. Furnaces in the system were set to 110 and 1000 degrees Celsius. The concentrations of Ag, As, Ba, Be, Bi, Cd, Ce, Co, Cr, Cs, Cu, Dy, Er, Eu, Ga, Gd, Ge, Hf, Ho, La, Lu, Nb, Nd, Ni, Pb, Pr, Rb, Sc, Sm, Sn, Sr, Ta, Tb, Th, Tl, Tm, U, V, W, Y, Yb, Zn and Zr were all determined by laser ablation ICP-MS on a fragment of the same glass disk used earlier for XRF analysis. Data quality was monitored by blind insertion of sample duplicates, internal reference materials, and the certified reference material OREAS 24b and blanks. Total uncertainties for major elements are ≤1.5%, those for minor elements are <2.5% (at concentrations >0.1 wt%) and those for most trace elements are ≤10% (Lu, ±20%).

### In-situ oxygen isotopes of zircon by SIMS

Zircon grains in samples LG2023B, LG2023A and LG2023C were analyzed for δ^18^O(VSMOW) (Vienna Standard Mean Ocean Water; ^18^O/^16^O = 0.0020052^69^,) in three analytical sessions (Supplementary Data 3) using a CAMECA IMS 1280-HR Secondary Ion Mass Spectrometer (SIMS) at the GFZ Helmholtz Centre for Geosciences, Potsdam. Zircon grains extracted from the hbl-TTG were mounted in the central part of one 25.4 mm diameter disk (>5 mm from the edges) along with the two zircon reference materials 91500 (δ^18^O = 9.86‰^70^,) and Temora-2 (δ^18^O = 8.20‰^71^). Polishing relief in the mount containing zircon separates was <5 μm, whereas it was larger (>10 µm) in both drill bit mounts, as confirmed by white-light profilometry. The epoxy discs were cleaned in high-purity ethanol and coated with 35 nm, high-purity gold coating needed to assure electrical conductivity during the analyses. A c. 2 nA ^133^Cs^+^ primary ion beam was focused to a 10 μm diameter spot with a total impact energy of 20 keV. A normal incidence electron gun was used for charge compensation. Prior to data collection, each analytical site was sputtered with a 20-μm raster for 70–80 s to remove the gold coat, suppress any surface contaminants and establish equilibrium sputtering conditions. Data collection used either a 15 or 10 μm raster to ensure a flat-bottom crater geometry. Each analysis was preceded by automatic centreing of the field aperture in X and Y, and the contrast aperture in X. Data were collected in multicollection mode using Faraday cup detectors. The count rates on the ^16^O^-^ mass station were typically 1.4 to 2.3 × 10^9^ counts per second (cps). The mass resolution of the SIMS (at 10% of peak height) was set at M/∆M ≈ 2000 during the first session and ≈5000 during the second and third sessions (enough to separate ^17^O^-^ and ^16^O^1^H^-^). Each analysis lasted c. 4 min including pre-sputtering, auto-centreing, and data acquisition routines, which consisted of 20 integrations of 4 s each. Additional details on analytical settings are given in the Supplementary Data 3 (SIMS metadata). The instrumental mass fractionation (IMF) was monitored by interspersed measurements throughout the analytical sessions in grains of the 91500-zircon reference material. No significant time-dependent drift was observed during any of the analytical sessions, which yielded a repeatability of ±0.09‰ (1 standard deviation, 1 s) in the zircon separate mount and ±0.26 to ±0.30‰ (1 s) in the drill bit mounts. The poor analytical precision in the drill bit mounts is attributable to the large surface relief, which has been previously documented to degrade the quality of SIMS oxygen isotope ratios analysis^72^. We used the Temora-2 zircon reference material as a quality control material to assess the accuracy of the analytical bias correction defined by the results from the 91500-reference material. In the zircon separate mount, the corrected δ^18^O values of Temora-2 was within 0.38‰ (outside the 2 s uncertainty of ±0.18‰ measured in 91500) from its published value, hence we consider this to be an estimate of the total uncertainty on the δ^18^O values measured in this mount. The corrected δ^18^O values of Temora-2 in the drill bit mounts were within 2 s uncertainty in 91500 (i.e., within 0.04 to 0.53‰) from the published value, and we believe that our data quality in these mounts is mostly controlled by the analytical precision (i.e. better than ~ ±0.30‰, 1 s).

During a fourth session zircon grains for sample LG2023-1 and LG2023-3 were analysed for δ^18^O at the SwissSIMS laboratory, Institute of Earth Sciences of the University of Lausanne (Switzerland). At SwissSIMS, the ^18^O/^16^O ratios were measured along with ^16^OH, using a CAMECA IMS 1280HR, with a 10 kV Cs^+^ primary beam and a ~ 2 nA current, resulting in a ~ 10 µm beam size. The electron flood gun, with normal incidence, was used to compensate charges. ^16^O, and ^18^O secondary ions, accelerated at 10 kV, were detected on L’2 and H’2 in multicollection using exit slit 1, and ^16^OH was detected simultaneously on axial FC, with exit slit set at 5000. Together with an entrance slit of 90 microns, it results in mass resolving power of 2400 for δ^18^O and 5000 for OH. Faraday cups are calibrated at the beginning of the session, using the calibration routine. Each analysis takes less than 5 minutes, including pre-sputtering of 90 s with a raster of 20 microns, to limit surface water contamination on the OH signal, followed by automated centreing of secondary electrons. This setting allowed a repeatability of 0.6‰ (2 standard deviations, SD) on a 91500 zircon reference material^70^ for the whole session, and a within-spot repeatability for each analysis usually ≤0.2‰ (2 standard error, SE). ^16^O^1^H^-^signal was only standardised to ^16^O to exclude OH variation resulting from primary beam intensity. No OH values are published for 91500, reported ^16^O^1^H^-^/^16^O are thus a qualitative measurement: higher ^16^O^1^H^-^/^16^O^-^ in unknowns compared to 91500 means higher OH concentration than crystalline 91500 zircon. The oxygen isotope compositions are expressed in δ notation and are referenced to the Vienna Standard Mean Ocean Water (VSMOW). All the analysis are listed in Supplementary Data 3.

### In-situ U–Pb geochronology of zircon

Zircon U–Pb measurements for sample LG2023B were collected at the GeoHistory Facility, JdLC, Curtin University, Perth, using a RESOlution M-50A-LR 193 nm ArF excimer laser system coupled to an Agilent 8900 triple quadrupole mass spectrometer. Laser beam diameter was set to 33 µm at a repetition rate of 10 Hz and an on-sample energy of ~2.3 J cm^-2^, with 20 s of background capture followed by 30 s of ablation. All analyses were preceded by two cleaning pulses. The sample cell was flushed by ultrahigh purity He (0.32 L min^-1^) and N_2_ (1.2 mL min^-1^).

The following masses were analysed (dwell time in ms): ^202^Hg (0.005), ^204^Pb (0.005), ^206^Pb (0.02), ^207^Pb (0.02), ^208^Pb (0.02), ^232^Th (0.02), ^235^U (0.02), ^238^U (0.02). Zircon U–Pb isotope data were collected on an Agilent 8900 triple quadrupole mass spectrometer with high purity Ar as the carrier gas (flow rate 0.98 L min-1). Analyses of ~20 unknowns were bracketed by analysis of a standard block containing the primary zircon reference materials GJ-1 (601.95 ± 0.40 Ma^73,74^;) and OG1 (3465.4 ± 0.6 Ma^75^), which were used to monitor and correct for mass fractionation and instrumental drift. The standard block also contained the Phanerozoic to Archaean reference materials Plešovice (337.13 ± 0.37 Ma^76^), 91500 (1063.78 ± 0.65 Ma^73,77^) and Maniitsoq (3008.70 ± 0.72 Ma^78^; all uncertainties at 2σ), which were used to monitor data accuracy and precision. During the analytical session, when reduced against a matrix-matched reference material, Plešovice, 91500 and Maniitsoq yielded statistically reliable (*p* > 0.05) weighted mean ages of 338 ± 5 Ma, 1058 ± 19 Ma, and 3008.9 ± 2.6 Ma, respectively, all of which are within uncertainty of the published age (see Supplementary Data 4 for full reference material compilation).

U–Pb isotopic data were reduced in Iolite4^79^. Uncertainties on analyses of primary reference materials were propagated in quadrature to the unknowns and secondary zircon reference materials. For the unknowns, some analyses ablated across inadvertent inclusions; these inclusion-related signals were cropped from the integrations or, if too large or numerous, integrations deleted; whilst the accuracy of the cropped analyses is maintained, the precision of the U–Pb data is significantly reduced due to the shorter integration times. Age calculations and plots were made using IsoplotR version 6.0^80^. All zircon dates are >1.5 Ga and are therefore presented as ^207^Pb/^206^Pb ages for optimum precision^81^. All uncertainties are presented at 95% confidence (2SE). Full isotopic data for the reference materials and unknowns are given in Supplementary Data 4.

Following the first U–Pb geochronology session at the John de Laeter Centre laboratory, additional spot analyses were collected for sample LG2023B to better characterise the age populations and hornblendite samples (LG2023A, LG2023C, LG2023-1 and LG2023-3). These additional analytical sessions included acquisition of selected trace element abundances and were run at the Institute of Petrology and Geochemistry at ETH Zürich. We ran a first session in October 2023 targeting zircon grains on mounts including LG2023B and LG2023A and C samples, a second session in December 2024 targeted polished thin section of samples LG2023-1 and LG2023-3 to identify additional older zircon for the hornblendite samples. Finally, a third session in May 2025 was carried out on the same samples LG2023-1 and LG2023-3 following oxygen isotope analysis using indium mount. For these three sessions zircon grains were analysed by laser ablation-inductively coupled plasma mass spectrometry (LA-ICPMS), employing a 193 nm Resonetics Resolution S155 laser ablation system coupled to a Thermo Element XR, sector-field single collector ICP-MS. The analytical setup, parameters and data reduction strategies are reported in Supplementary Data 5, following community-derived guidelines^73^.

U–Pb isotopic data were reduced in Iolite4^79^. Uncertainties on analyses of primary reference material were propagated in quadrature to the unknowns and secondary zircon reference materials. Zircon analyses are considered concordant where the error ellipses at 2σ generated by the ^207^Pb/^206^Pb and ^206^Pb/^238^U ratios overlap the inverse concordia curve, excluding uncertainties on the decay constant. Age calculations and plots were made using IsoplotR version 6.0^80^. All zircon dates are >1.5 Ga and are therefore presented as ^207^Pb/^206^Pb ages for optimum precision^81^. All uncertainties are presented at 95% confidence (2SE). For trace element quantification we used stochiometric Si or alternatively Zr content in zircon as internal standard (15.2 wt% Si and 43 wt% Zr), and SRM NIST610 as external CRM. Ti was quantified by zircon 91500 (Ti: 4.73 ± 0.15 mg g^−1^) as suggested by ref. ^82^. Full isotopic and trace elements data for the reference materials and unknowns are given in Supplementary Data 5.

### In-situ Hf isotopes of zircon

Zircon Hf isotopic analyses for hornblendite sample LG2023C were carried out by laser ablation—multi-collection—inductively coupled plasma—mass spectrometry (LA-MC-ICP-MS) at ETH Zürich using a RESOlution (ASI/Applied Spectra) excimer ArF (193 nm wavelength) laser ablation system equipped with the dual-volume S-155 ablation cell (Laurin Technic), attached to a Nu Plasma 2 (Nu Instruments), multi-collector sector-field mass spectrometer. A laser spot size of ~50 μm, a repetition rate of 5 Hz and an energy density of ~4 J cm^–2^ were used for static spot analyses. The carrier gas consisted of high-purity helium (c. 0.35 L min^−1^) and argon sample gas from the MC-ICP-MS (c. 1.0 L min^−1^). The MC-ICP-MS was optimised for maximum sensitivity on Hf isotopes and peak alignment throughout the investigated mass range. We acquired intensities for the following isotopes (corresponding Faraday cup indicated between brackets): ^171^Yb (L4), ^173^Yb (L2), ^175^Lu (Ax), ^176^(Yb + Lu + Hf) (H1), ^177^Hf (H2), ^178^Hf (H3), ^179^Hf (H4), ^180^Hf (H5), ^181^Ta (H6). The data were processed offline with the software Iolite4^79,83^, using an inhouse data reduction scheme. Instrumental mass bias for Yb and Hf isotopes were corrected to the natural abundance ratios ^173^Yb/^171^Yb and ^179^Hf/^177^Hf and the isobaric interferences of ^176^Yb and ^176^Lu on 176Hf were corrected using the natural abundance ratios of ^176^Yb/^173^Yb and ^176^Lu/^175^Lu, with all natural abundance ratios taken from^84^. The mass bias correction factor obtained for Yb isotopes was applied to Lu isotopes. Accuracy and external reproducibility of the method were controlled by repeated analyses of reference zircon standards Mud Tank^85^, GJ-1^86^, Plešovice^76^, GHR1^87^ and Temora^85^. The ^176^Hf/^177^Hf ratios obtained on all reference materials are within uncertainties identical to the recommended values. The initial ^176^Hf/^177^Hf ratios were calculated for each analysis using a ^176^Lu decay constant of 1.865 × 10^–11^ ^88^, the measured ^176^Lu/^177^Hf ratio and U–Pb crystallisation ages previously constrained by LA-ICP-MS on same zircon domains. The initial εHf were calculated using the parameters for the chondritic uniform reservoir (CHUR) recommended by ref. ^89^. The quoted uncertainties on initial isotopic compositions of unknowns include the analytical uncertainty (2 S.E.) and the average intra-session reproducibility (2 S.D.) of initial isotopic compositions of reference materials (typically ~1 εHf unit), propagated by quadratic addition.

For Hf isotope measurements of hbl-TTG sample LG2023B, a Thermo-Scientific NEPTUNE Plus multicollector (MC-ICP-MS system at the Frankfurt Isotope & Element Research Center (FIERCE) of the Goethe University Frankfurt (Germany), coupled to a RESOlution 193 nm ArF Excimer laser system with S155 two-volume ablation cell was used. The analytical method including data processing was described in refs. ^90,91^. Laser spots of 60 µm diameter used for Lu–Hf isotope analyses were superimposed directly on the U–Pb laser spots. The results of Hf isotope analyses are shown in Fig. 4 and in Supplementary Data 6, including results of standard measurements. Each grain was ablated for 36 s using a fluence of 3.5 J cm^−2^ and a frequency of 5 Hz. Standard measurements yielded the following interference, mass bias, and offset corrected ^176^Hf/^177^Hf ratios: GJ1 (primary standard used for offset correction) = 0.282667 ± 0.000030 (2 S.D., standard deviation, *n* = 35); and Temora2: 0.282669 ± 0.000042 (*n* = 99) in good agreement with published data^85,86^.

## Supplementary information

Supplementary Information

Description of Additional Supplementary Files

Supplementary Data 1

Supplementary Data 2

Supplementary Data 3

Supplementary Data 4

Supplementary Data 5

Supplementary Data 6

Transparent Peer Review file

## Data Availability

All data supporting the findings of this study are presented in the manuscript and Supplementary Information and Data. These data are also available at the ETH repository: 10.3929/ethz-c-000797340.
