# Supplementary material for: Insights into early continental crust formation from the most ancient heart of Scotland
Source: Nat Commun. 2026 Apr 21;17:5474. doi: 10.1038/s41467-026-72076-6 (PMC13284193; doi:10.1038/s41467-026-72076-6)
Supplement: Supplementary file 1 — Supplementary Information [file 41467_2026_72076_MOESM1_ESM.pdf]

Supporting Information for

Insights into early continental crust formation from the most ancient heart of Scotland

Silvia Volante[1]*, Fernanda Torres-Mancinelli[1,2], Jonas Kaempf[3], Vitor Barrote[4], Tim Johnson[3], Christopher Kirkland[3], Maria Rosa Scicchitano[5], Lorenzo Tavazzani[6], Sampriti Basak[7], Anne-Sophie Bouvier[8], Annika Dziggel[2], Axel Gerdes[9]

[1] Department of Earth and Planetary Sciences, Structural Geology and Tectonics Group, Geological Institute, ETH Zürich, Zürich, 8092, Switzerland

[2] Department of Tectonics and Resources, Institute of Geoscience, Ruhr–Universität Bochum, Bochum, 44801, Germany

[3] Curtin Frontier Institute for Geoscience Solutions, School of Earth and Planetary Sciences, Curtin University, Perth, WA 6845, Australia

[4] Center for Nuclear Engineering and Sciences, Paul Scherrer Intitute, Villigen, 5232, Switzerland

[5] GFZ Helmholtz Centre for Geosciences, Potsdam, 14473, Germany

[6] Department of Earth and Planetary Sciences, Institute of Geochemistry and Petrology, ETH Zürich, Zürich, 8092, Switzerland

[7] Department of Geosciences and Natural Resource Management, University of Copenhagen, Copenhagen, 1350, Denmark

[8] Institut des Sciences de la Terre, Université de Lausanne, Quartier UNIL-Mouline, Lausanne, 1015, Switzerland

[9] Department of Geosciences, Frankfurt Isotope and Element Research Center (FIERCE), Goethe-University Frankfurt, Frankfurt, 60438, Germany

*svolante@eaps.ethz.ch

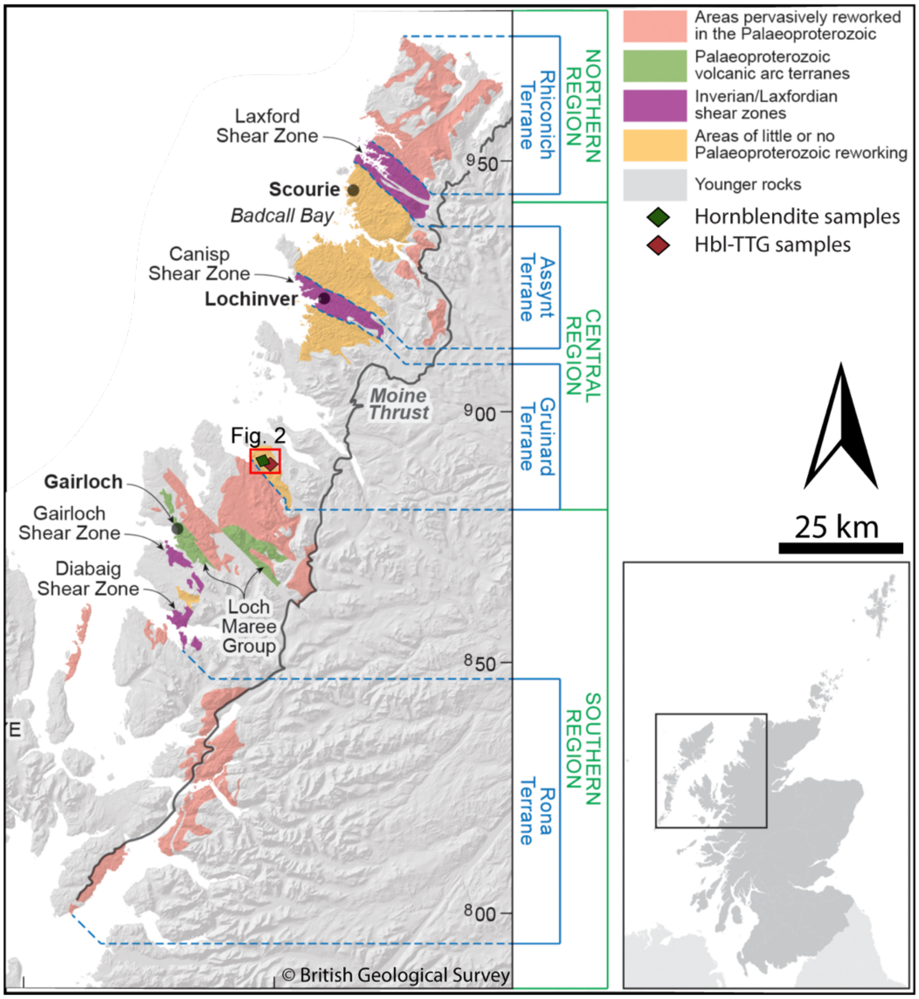

Supplementary Figure 1. | Simplified geological map of the mainland Lewisian Gneiss Complex, NW Scotland. Map modified after (1) shows the location of the samples investigated in this study. Derived from BGS Geology 50k Digital Data Licence No. 2021/018 British Geological Survey © and Database Right UKRI. All rights reserved.

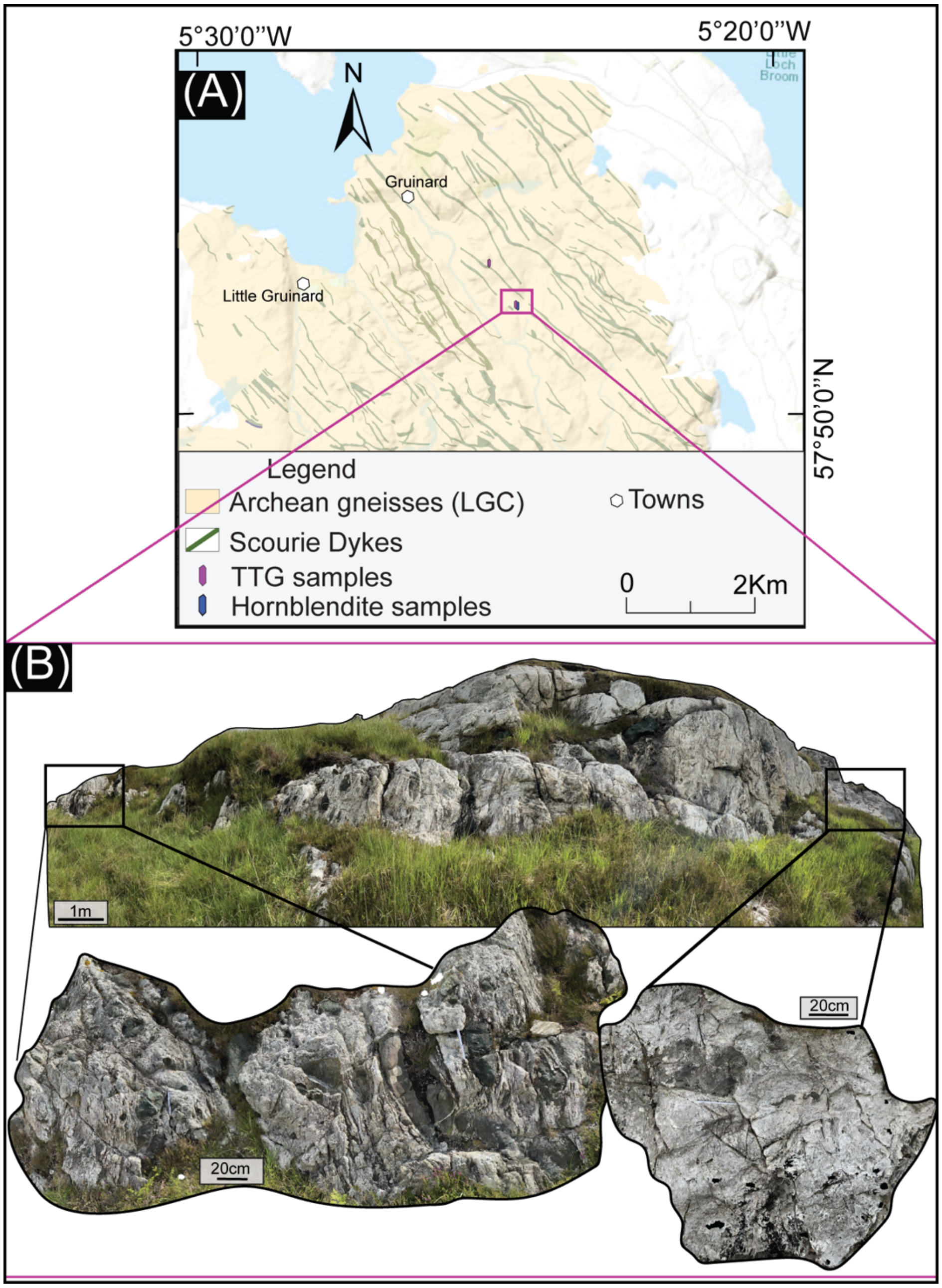

Supplementary Figure 2. | Geological map and field observations of the study area in the Gruinard Terrane of the LGC. (A) Simplified geological map of the study area. Map modified after (1) shows the location of the samples investigated in this study. Derived from BGS Geology 50k Digital Data Licence No. 2021/018 British Geological Survey © and Database Right UKRI. All rights reserved. **(B)** Outcrops dominated by hbl-TTG with enclosed hornblendite pods, where the investigated samples were collected.

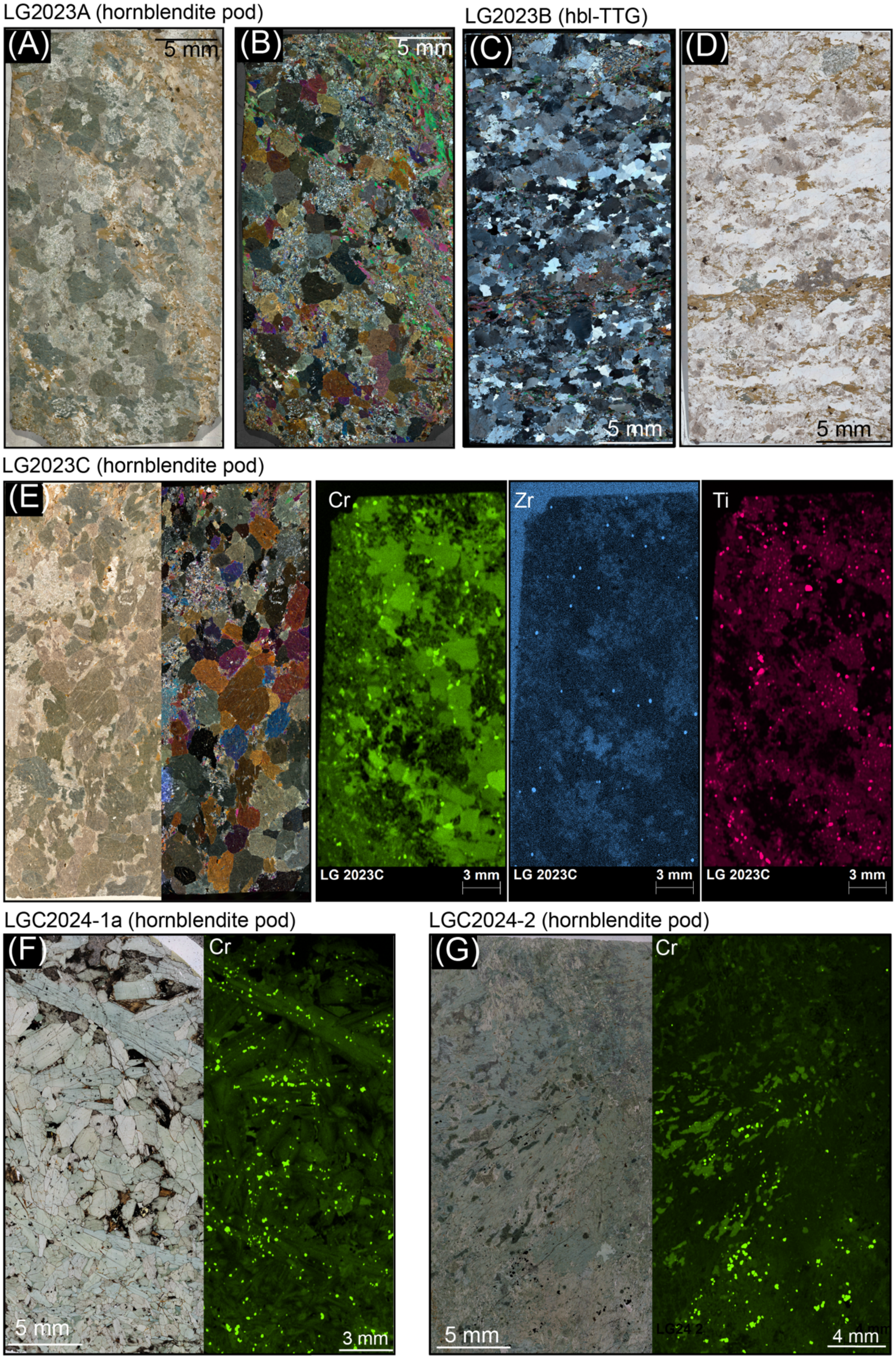
LG2023A (hornblendite pod)
LG2023B (hbl-TTG)
(A)
5 mm
(B)
5 mm
(C)
5 mm
(D)
5 mm
LG2023C (hornblendite pod)
(E)
Cr
Zr
Ti
LG 2023C
3 mm
LG 2023C
3 mm
LG 2023C
3 mm
LGC2024-1a (hornblendite pod)
LGC2024-2 (hornblendite pod)
(F)
Cr
5 mm
3 mm
(G)
Cr
5 mm
4 mm

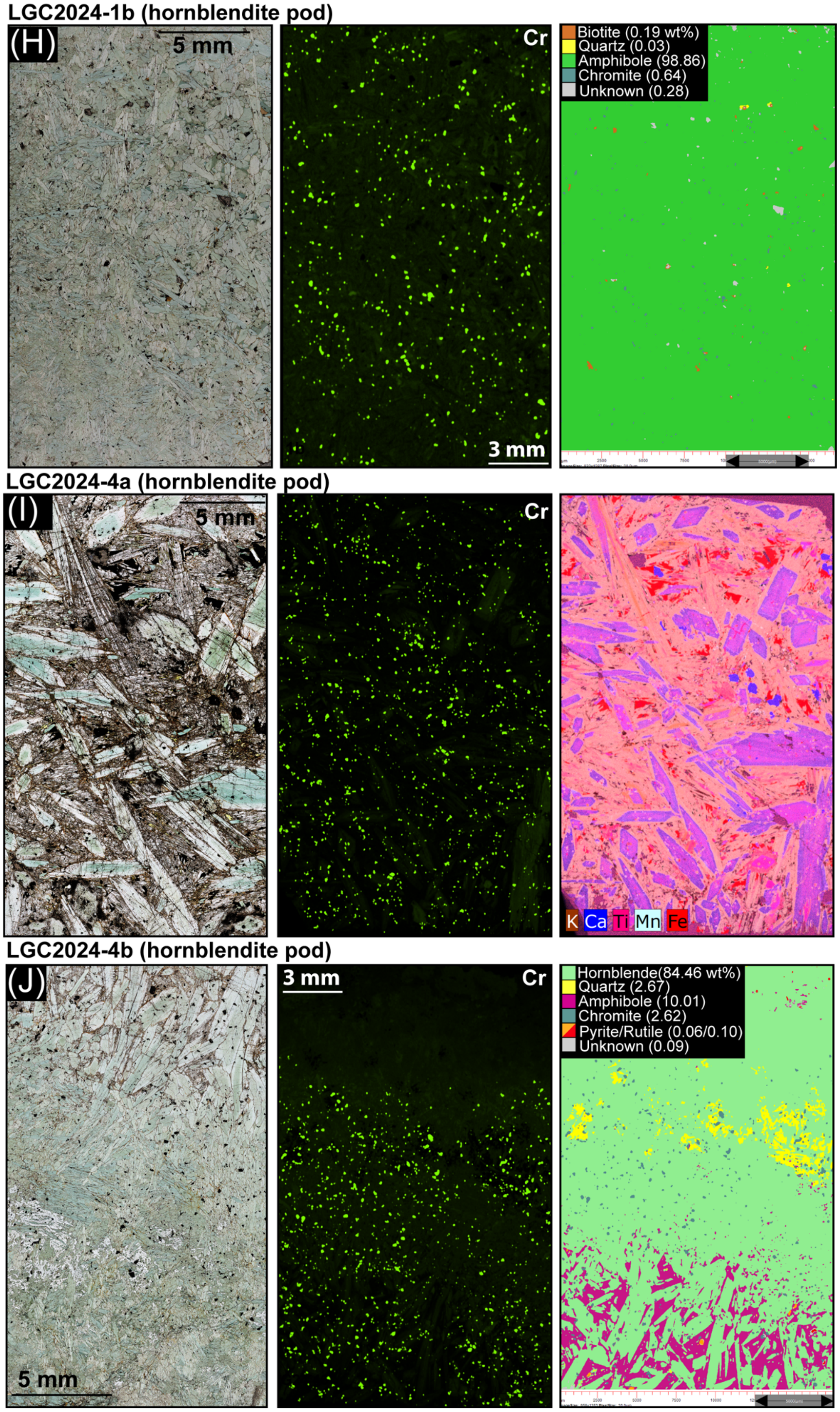
LGC2024-1b (hornblendite pod)
(H)
5 mm
Cr
3 mm
Biotite (0.19 wt%)
Quartz (0.03)
Amphibole (98.86)
Chromite (0.64)
Unknown (0.28)
LGC2024-4a (hornblendite pod)
(I)
5 mm
Cr
K
Ca
Ti
Mn
Fe
LGC2024-4b (hornblendite pod)
(J)
5 mm
3 mm
Cr
Hornblende(84.46 wt%)
Quartz (2.67)
Amphibole (10.01)
Chromite (2.62)
Pyrite/Rutile (0.06/0.10)
Unknown (0.09)

Supplementary Figure 3. | Thin Section and micro-XRF elemental maps of the analysed samples. (A) plane and **(B)** cross polarized light microphotographs for the investigated hornblendite sample. **(C)** Cross and **(D)** plane polarized light microphotographs for the investigated hbl-TTG sample. **(E)** Optical imaging and micro-XRF elemental maps of Cr, Zr and Ti for the hornblendite sample. **(F–G)** Representative optical images and Cr-elemental map for hornblendite pods. **(H–J)** plane polarized light microphotographs, Cr-elemental map, phase and elemental maps for three hornblendite pods.

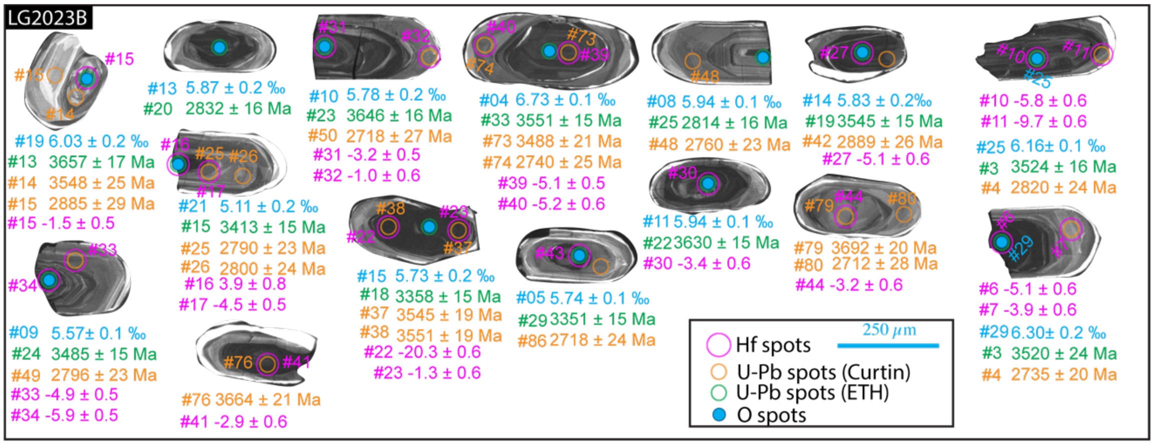

Supplementary Figure 4. | **Cathodoluminescence (CL) images of zircon from the analysed hbl-TTG.** Representative CL images of zircon grains for the studied hbl-TTG sample.

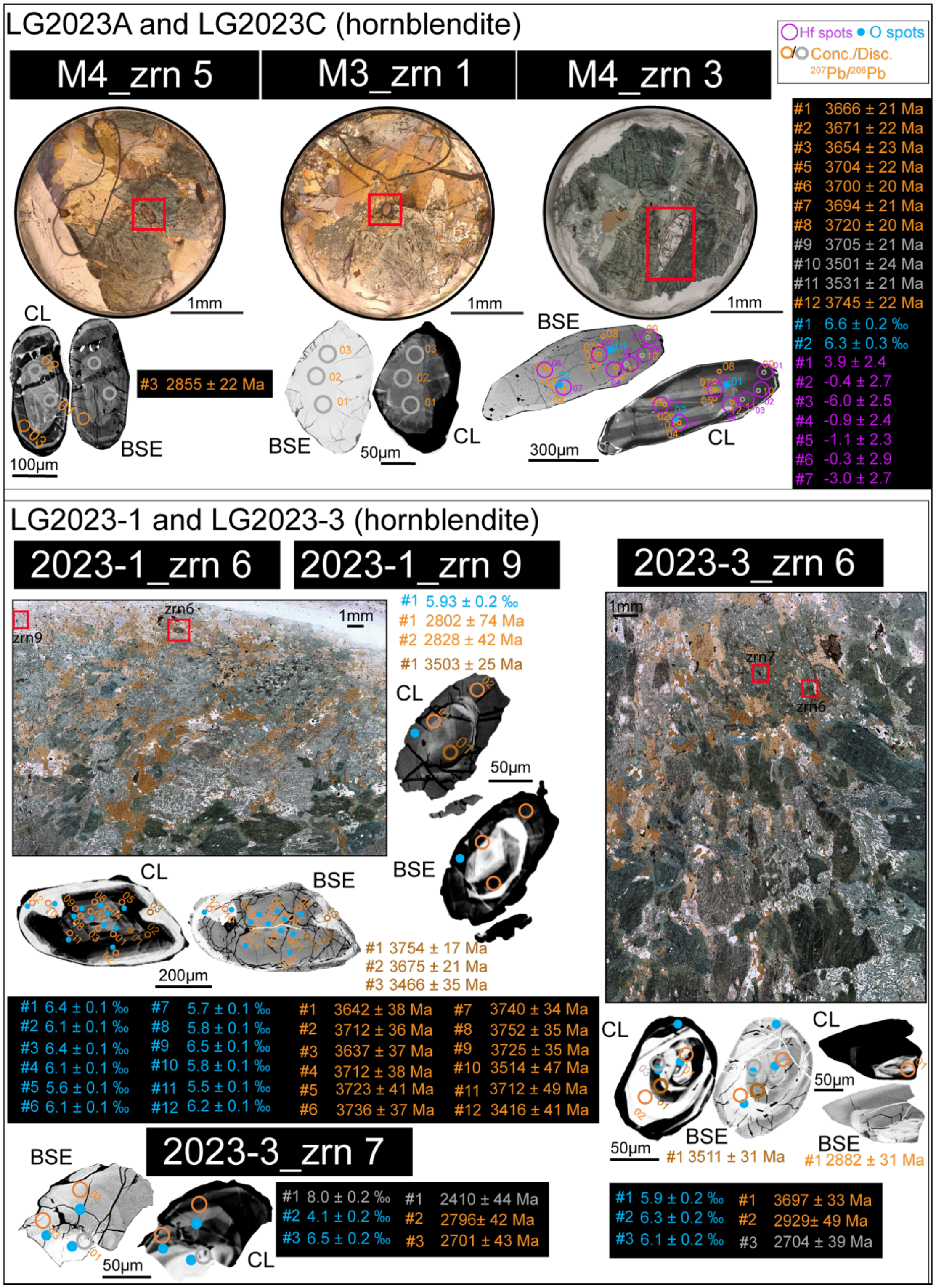

Supplementary Figure 5. | Optical, cathodoluminescence (CL), and back-scattered electron (BSE) images for the analysed zircon grains in the hornblendite sample. For each analysed zircon grain U-Pb, Hf and O spot analysis are reported.

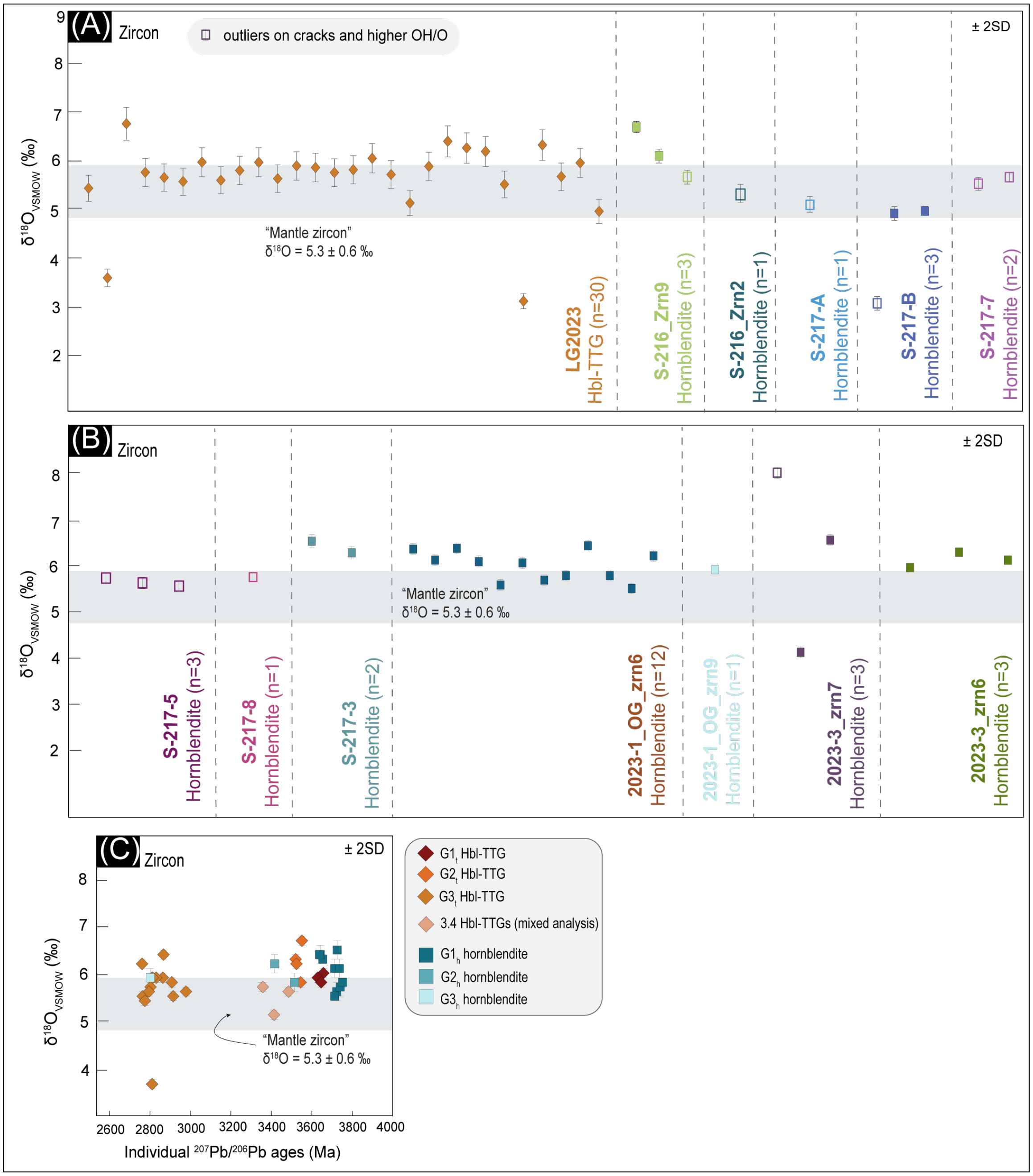

Supplementary Figure 6. | Oxygen-isotope compositions for the investigated samples. (A–B) Individual oxygen isotope analyses of zircon for the investigated samples. **(C)** Individual Oxygen-isotope compositions color-coded by age population.

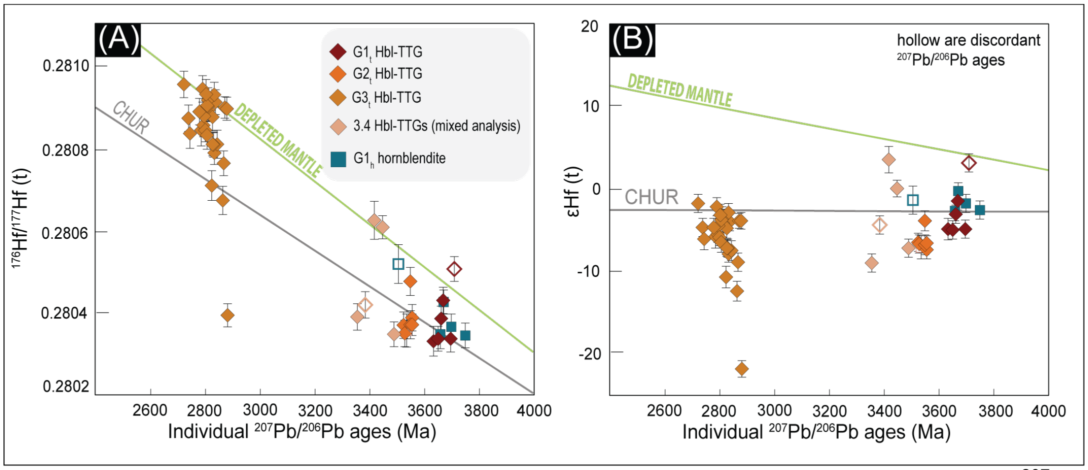

Supplementary Figure 7. | Zircon initialHf and εHf$_{(t)}$ isotope versus individual spot ^{207}Pb/^{206}Pb dates (in Ma) for the investigated samples. (A) Zircon ^{176}Hf/^{177}Hf$_{(i)}$ versus individual spot dates (in Ma) of the same samples showing the horizontal trends characteristic of Pb-loss combined with undisturbed Hf isotopes. **(B)** Zircon εHf$^{(t)}$ versus individual spot ^{207}Pb/^{206}Pb dates (in Ma) illustrating the effect of Pb-loss and/or unsupported radiogenic Pb on initial Hf signatures.

Supplementary Data 1 (separate file). Complete list of samples investigated in this study.

Supplementary Data 2 (separate file). Bulk-rock major (wt%) and trace element (ppm) compositions for the investigated samples.

Supplementary Data 3 (separate file). Oxygen isotope results.

Supplementary Data 4 (separate file). U–Pb geochronology of zircon carried out at Curtin University.

Supplementary Data 5 (separate file). U–Pb geochronology of zircon carried out at ETH Zürich.

Supplementary Data 6 (separate file). Hf isotope results.

SI References

1. S. Volante, *et al.*, Constraints on the Paleoproterozoic tectono-metamorphic evolution of the Lewisian Gneiss Complex, NW Scotland: implications for Nuna assembly. *Journal of Metamorphic Geology* Accepted (2023). https://doi.org/10.1002/JMG.12748.
