# Supplementary material for: Insights into early continental crust formation from the most ancient heart of Scotland
Source: Nat Commun. 2026 Apr 21;17:5474. doi: 10.1038/s41467-026-72076-6 (PMC13284193; doi:10.1038/s41467-026-72076-6)
Supplement: Supplementary file 2 — Description of Additional Supplementary Files [file 41467_2026_72076_MOESM2_ESM.pdf]

Description of Additional Supplementary Files

Supplementary Data 1. Complete list of samples investigated in this study.

Supplementary Data 2. Bulk-rock major (wt%) and trace element (ppm) compositions for the investigated samples.

Supplementary Data 3. Oxygen isotope results.

Supplementary Data 4. U–Pb geochronology of zircon carried out at Curtin University.

Supplementary Data 5. U–Pb geochronology of zircon carried out at ETH Zürich.

Supplementary Data 6. Hf isotope results.
