# Supplementary material for: Insights into early continental crust formation from the most ancient heart of Scotland
Source: Nat Commun. 2026 Apr 21;17:5474. doi: 10.1038/s41467-026-72076-6 (PMC13284193; doi:10.1038/s41467-026-72076-6)
Supplement: Supplementary file 9 — Transparent Peer Review file [file 41467_2026_72076_MOESM9_ESM.pdf]

nature portfolio

Peer Review File

Insights into early Earth continental crust formation from the most ancient heart of Scotland

Corresponding Author: Dr Silvia volante

This file contains all reviewer reports in order by version, followed by all author rebuttals in order by version.

Version 0:

Reviewer comments:

Reviewer #1

(Remarks to the Author)
First, I would like to disclose that I have previously worked with some of the authors during my time at Curtin University several years ago. While this may be considered by some as a potential conflict of interest, I decided to take that and provide an objective and fair review.
The authors present new whole-rock geochemical data, along with zircon U-Pb, O, Hf, and trace element analyses, from two TTG samples (??) and seven hornblendite samples. The study reports the discovery of a ca. 3.7 Ga TTG–(ultra)mafic rock association, which represents a significant and exciting finding for the early Earth research community. Notably, the authors suggest that the hornblendite (ultra)mafic rocks may be relics of the early mafic crust.
While the analytical data presented are of high quality, I find the discussion on the origin and petrogenesis of both the TTG and hornblendite suites to be limited and in need of further elaboration. The TTG interpretations are particularly constrained by the small number of samples (only two), which warrants more careful contextualization.
An intriguing observation is that the oldest zircon population in the hornblendites appears to be slightly older than (with high-resolution separation of the ages) Group 1 zircons in the TTG samples. This raises the possibility that the (ultra)mafic rocks may have served as a source for the TTGs, even those rocks found as xenoliths within TTG rocks, which highlights the assimilation later. I encourage the authors to explore this hypothesis further, potentially through detailed geochemical modeling and thermodynamic simulations.
Additional minor comments:
• Line 195 & 212: The authors mention "zircon growth in a water-rich environment." Please elaborate on this point and consider citing relevant literature to support the interpretation.
• Line 133: It would be helpful to include Eu* values in the discussion of geochemical characteristics.
• Line 116: Consider comparing your hornblendite data with average compositions of both depleted and enriched Archean tholeiitic basalts.
• Line 57: Please correct “meteoric” to “meteorite.”
Regards,
Hamed Gamaleldien

Reviewer #2

(Remarks to the Author)
Dear Authors

This is a surprising and exciting discovery. I was certainly not expecting TTGs and associated hornblendites of this age to crop out in the Lewisian and I think the dual ages of these components is certainly a big deal and worthy of publication in a widely read journal.

You have clearly given location lat and long for the samples in the appendices, which is good. I wonder if a simple geological map in the appendices could be added as I’m quite sure that many would like to see that too.

I do think there could be a bit more clarity on the interpretation of the G1 and G2 ages from both TTG and hornblendite. It is not entirely clear whether you are invoking the former as being intrusion ages and the latter as magmatic or metamorphic

ages. Is the simplest explanation that the G1 ages are intrusive and the latter are metamorphic? Why are you being circumspect on this? I also wonder if you have tried some zircon trace elemental ratios other than Th/U to try and elucidate the source e.g. Hf vs U/Yb? This might also add to the debate on mantle vs crustal input.

For the Hf isotopes, an obvious question where you have datasets with U-Pb ages that are spread and have some discordance is whether you have performed discordance tests against 176Hf/177Hf and EHf as that could account for some of the spread in data that you explain in a slightly complex way regarding sources and input of later juvenile additions vs much later reworking in the Neoarchaean. I would personally recommend including such a figure in the supplementary files along with a few words discounting (if that's provable) this as a mechanism for some of the spread in Hf isotopes in the G1 and G2 zircons.

The O isotope story is intriguing, but I think plotting all zircons rather than just median values might be useful. In doing this you have implied that the Neoarchaean zircons are more mantle like than the Eoarchaean ones from which surely you are reworking material? This would require Neoarachean mantle input which is juvenile but has Eoarchaean reworked crustal material in its source and therefore that the TTG actually has a Neoarchaean intrusive age? This does have a bearing on your interpretation of the older zircons as being from a wetter source so I think there should be internal consistency in your argument. I note also that you don't have any Hf for G3 hornblendite zircons which might have shed some light on the relationship between these two different lithologies and their evolution in Neoarchaean times. Is this because the rims were too small? I think this is worthy of mention.

In the supplementary files I think the data are overall transparent. I do think that some plots for the U-Pb RMs in S4 and S5 would be useful. Also, in S5, the ETH spreadsheet does quantify total external uncertainty at 1% for all RMs plus 0.5% long term excess, which are propagated in quadrature to all unknowns. I wonder where these figures come from as they are all the same and seem more like an estimate rather than being strictly quantified. Could you comment on this in the methods? I also noted that in S6 in the FIERCE Frankfurt RMs worksheet that you have labelled the second set of zircons as GJ-1 (cell A48) but these have an assigned age of 417 Ma so either the age is wrong or you have mislabelled Temora as GJ-1?

Overall, this is an exciting discovery and is supported by good data. The ms is written well with good figures and clear supplementary files. I hope my comments might help you to improve a bit further the paper with not much extra work.

Best wishes
Craig Storey

Reviewer #3

(Remarks to the Author)
The manuscript claims to provide evidence for the preservation of amphibolitized Hadean crust as enclaves in Eoarchean TTG gneiss. The mere discovery of an Eoarchean component in the Lewisian gniesses, supporting hypothetical associations with the North Atlantic Craton in the Archean, is an important discovery on a local scale. However, the paper is more ambitious than that, and seeks to establish that preserved Hadean mafic protocrust is preserved, in similar fashion to the contested claims of Hadean faux-amphibolite in far northeastern Canada have done. However the evidence in comparison is limited to the interpretation of small pods of amphibolite embedded in felsic gneiss, unlike the substantial tracts of mafic rocks found at Nuvvuagittuq.
Firstly, it is just misleading to claim that the hornblendites "represent vestiges of a primitive hydrated (proto)crust", when the oldest zircon age estimate is only 3.7Ga and model ages along a 'default' depleted mantle array range from 4.1 to 3.9Ga. Without careful reading, this gives the impression that the rocks themselves are Hadean, which the authors know is not true. Beyond the assumption that such an assumed mantle array requires continual crustal growth from the beginning of the Earth that approximates in composition modern continental crust, an idea whose time has long since passed, a vague estimate for mantle derivation around 4Ga is hardly evidence that the crustal source of melting is older than 4Ga, let alone the age of the amphibolite itself.
A basic problem in the chain of logic can be found simply by looking at the photographic evidence – these enclaves are not simply amphibolites in the strict IUGS definition, but coarse-grained mesocratic mafic pods that are not properly described in sample description section – here the authors merely mention the presence of amphibole, whereas the field and microphotos show pods with an abundance of felsic minerals along with amphibole, which they vaguely describe as 'pegmatitic' without explanation. These pods have reaction mantles with the host TTGs that are almost monomineralic (hornblende with minor biotite and very little plagioclase or quartz), a strong indication that the assemblage is a product of metasomatic interaction with the granitoid host. This is supported by the chemical maps provided, which demonstrate mineralogical and chemical banding absolutely characteristic of reaction textures during metamorphism. That mny pods are entirely hornblendite merly indicates that the original encvlave has been consumed during metamorphism. As such, interpreting these as some kind of 'primordial' amphibolite is highly suspect. Instead, the textures are entirely consistent with metamorphic mineral growth from basaltic, doleritic or even gabbroic xenoliths in a granitoid, with hydration accompanying metamorphism. If the zircon have only been found in the hornblendite rinds, and not in the 'pegmatitic' cores, it is possible that they all derive from the TTG host. The in-situ hosts of grains are nonetheless mafic, and the well-documented presence of zircon within them is important and needs explanation.
The coarse, complex character of the grains fits with an association with a gabbroic or granitoid protolith, rather than just growth with the monomineralic hornblendite during metamorphism. The diversity in age texture and composition is attributed by the authors to multistage modification of originally magmatic zircon of ca. 3.7 age. However, not all the variability is easily relegated to this cause.

It is claimed that both internal zircon textures and Th/U are used to discriminate between generations of zircon growth. There is no evidence presented in either figure or text that this is the case – we are left to look at the handful of grain images provided and work out how they were interpreted ourselves. The documentation of relations between analyses and spot positions is better for the TTG but is obscured for the critical amphibolite data. All Hf data come from a single grain, which yielded variable Pb-Pb ages. The assumption made that age variation is a product of disturbance during metamorphism – and hence all Hf isotopic values should be calculated for the oldest age 'population' – can be tested by the variation in eHf that results: low variability would confer validity to the assumption. Instead, eHf scatters above and below chondritic values, invalidating the assumption. Isotopically, the diversity in eHf measurements at 3.7Ga is a red flag- it points to either multiple, poorly mixed sources of Hf or the erroneous assignment of all data to a single Eoarchean age. It certainly cannot be attributed to a single, so called "Hadean" depleted mantle source, nor could the higher values be derived from anything older than the late Eoarchean. The fact that all analyses come from a single grain even calls into question whether the zircon is magmatic or contains a xenocrystic component not associated with the host protolith.
The slightly heavier oxygen isotope values from the amphibolite may seem meaningful if the dta from this study is taken by itself, but is well within the variation commonly seen in Eoarchean magmatic protoliths in the literature. As such there is no significant meaning in the variation seen.
There is important work here, carefully documented. It has importance for the relationship between Scotland and other Archean components of the North Atlantic Craton. However the authors have chosen to use good data stretched thinly over a frame thin interpretation. A more humble approach would make this a great paper.

Other comments
On line 140-1 there isa claim to support interpretation with texture and Th/U – yet there is nothing in the diagrams referred to support such a claim.
The Group one pooled age for LGC2023A is misleading – an MSWD of almost 5 for 18 data is a clear failure of the test of equivalence. This is partly due to the inclusion of one older outlier - the remaining data are to the age derived from the host TTG. Thus the interpretation of an older amphibolite in a pre-metamorphic TTG host rock is biased by one data point. It does not eliminate the possibility that zircon in the mafic enclave is actually from the TTG host.
The slightly heavier oxygen isotope values from the amphibolite may seem meaningful if the dta from this study is taken by itself, but is well within the variation commonly seen in Eoarchean magmatic protoliths in the literature. As such there is no significant meaning in the variation seen. For oxygen isotope values near the upper mantle limit ($\delta^{18}O$ of ca. 5.9 ‰) the authors invoke interaction between "surface water" and magma, but do not specify the isotopic composition of such water (is it SMOW?). In fact, surface-derived water commonly has sub-mantle values ($\delta^{18}O < 4.9$ ‰) and meteoric water has $\delta^{18}O$ as low as –30 ‰. High-temperature interaction with low- $\delta^{18}O$ waters would lower primary, mantle-like $\delta^{18}O$ rather than elevate it. By contrast, crustal materials that interacted with surface water, for example sediments, acquire supra-mantle $\delta^{18}O$. An easier explanation for the elevated zircon $\delta^{18}O$ is assimilation of such sediments into the magma.

The authors do not clearly state in the manuscript the true age of these TTG rocks nor whether the ca. 3.64 and ca. 3.52 Ga zircon populations are inherited/xenocrysts or represent the protolith crystallization age. The ages of ca. 2.8 Ga were also obtained from oscillatory-zoned domains which include older cores suggesting inheritance. In the Fig. 2C, a substantial number of analyses plot discordantly towards ca. 500 Ma on the concordia diagram. This should be also commented on in the text.
In-line comments:
L30: add the information about the age of TTG. Also, the two felsic samples are tonalitic, not "TTG"
L35-36: $\delta^{18}O$ of 6.3 ‰ does not require contribution of Hadean continental crust — so it's better to move this information after εHf. Also elevated $\delta^{18}O$ requires interaction with crustal material itself rather than surface-derived water.
L92: Here the authors state that hbl-TTG contains plagioclase, quartz and hornblende, while in L98 state that it contains hornblende, plagioclase and quartz. Then, which is the most common mineral in these two hbl-TTGs?
L104-105: In Figs. 1D,E the studied "TTG" are more similar to sanukitoids. Can authours add an comment on that?
L187: How the presence of a juvenile component support the interpretation of G1 and G2 as magmatic relics?
L273-274: The authors state "interaction between surface water and magmatic processes". Surface water can interact with magmas or during magmatic processes.
L277: The term "low-temperature oxygen" is a shortcut and should be omitted. Please use "exchanged oxygen isotopes with hydrosphere at low temperatures".
Fig. 2F: "No residual plagioclase" should point to Sr, not Nd in the figure.
Fig. 3B: it should spell "(Ce/Yb)N" on the y axis. This figure is not informative as the values of (Ce/Yb)N of the three zircon groups are similar. I propose to use age vs. Th/U – which is also commented in the text.
Fig. S2: Does really the maps show weight percent? Not area percent?

Reviewer #4

(Remarks to the Author)
I co-reviewed this manuscript with one of the reviewers who provided the listed reports. This is part of the Nature Communications initiative to facilitate training in peer review and to provide appropriate recognition for Early Career Researchers who co-review manuscripts.

Version 1:

Reviewer comments:

Reviewer #1

(Remarks to the Author)
After carefully reading the revised version, I would like to thank the authors for their excellent work in addressing nearly all of the comments from the other reviewers and me. The revision has significantly improved the manuscript, providing valuable insights into the Early Earth's crustal evolution and associated magmatism. The dataset is new and of high quality, and the study is certainly worthy of publication.
Best of Luck!
Hamed Gamaleldien

Reviewer #2

(Remarks to the Author)
Dear Authors

I think you have done a thorough job of responding to the reviews and have clearly shown where any changes have been made. I feel that the manuscript is stronger as a result and it is in good shape. Congratulations on this excellent discovery and the attention it will bring as a result. It's great to see the Lewisian bringing new and unexpected insight. I do not have any further recommendations or corrections.

Best wishes
Craig Storey

Reviewer #5

(Remarks to the Author)
This manuscript presents new geochemical and isotopic data from hornblende-bearing TTGs and associated hornblendite pods from the Lewisian Gneiss Complex, and reports zircon populations older than 3.6 Ga. These data potentially address an important question concerning the formation of early continental crust and the preservation of ancient crustal components within the North Atlantic Craton. The study combines petrographic observations, whole-rock geochemistry, and in situ zircon geochronology and isotopic analyses, which together may provide useful constraints on early crustal processes.

However, despite the revisions made in response to previous reviews, the manuscript still suffers from substantial problems in structure, clarity, and scientific presentation. In its current form, the text is often difficult to follow and does not consistently adhere to common practices of scientific writing. These issues make it challenging to evaluate the scientific interpretations presented in the paper.

The main concerns are summarised below.

1. Structure and organisation

The manuscript would benefit from a clearer separation between description, results, and interpretation. In several sections, particularly the Results and Discussion, descriptive observations, numerical data, and interpretation are interwoven. As a result, it is not always clear which statements represent observations and which represent interpretation, nor which interpretations are proposed by the authors and which are derived from previous studies. In addition, parts of the Discussion repeat descriptive information already presented in the Results rather than focusing on interpretation and synthesis.

2. Presentation of results

The Results section contains long passages listing numerical values, ratios, and sample identifiers directly in the text. This substantially reduces readability. Detailed numerical data and sample identifiers would be more effectively presented in tables, while the text should focus on highlighting patterns, contrasts, and relationships within the dataset.

3. Clarity of writing

Many sentences are long and syntactically complex, which obscures the main message of the text. Simplifying sentence structure and breaking complex statements into shorter sentences would significantly improve readability and make the scientific argument easier to follow.

4. Discussion

The Discussion remains largely descriptive and does not consistently develop a clear line of reasoning from the data to the proposed interpretations. Results are sometimes restated rather than interpreted, and interpretative statements are occasionally introduced without clearly explaining how they follow from the observations. A more focused Discussion that explicitly links observations, interpretations, and broader implications would strengthen the manuscript.

5. Framing of the research question

While the Introduction provides useful background, the specific research question and objectives of the study could be articulated more clearly. A clearer formulation of the aims would help guide the reader through the Results and Discussion.

Summary

The dataset presented in this manuscript may provide valuable insights into early crust formation in the Lewisian Gneiss Complex. However, substantial revision is required to improve clarity, structure, and adherence to standard practices of scientific writing. In particular, the separation between Results and Discussion should be clarified, detailed numerical data should be moved to tables where appropriate, and the overall narrative should be streamlined to emphasise the key observations and their implications.

Addressing these issues would greatly improve the readability of the manuscript and allow a clearer assessment of the scientific significance of the work.

Detailed comments
Lines 36-38: Question - What are the structural relationships between the TTGs and the preserved vestiges in the field?
Lines 42-43: The TTGs record not only deformation and metamorphism but also preserve primary structures related to partial melting.
Lines 63-70: The original formulation is slightly confusing because both clauses refer to extraterrestrial bodies. The paragaph could be rephrased for clarity, for example: "The origin of Earth’s hydrosphere has long been explained by the delivery of water by comets and asteroids. However, a growing body of evidence suggests that much of the water may have been present earlier, sequestered within the numerous bodies that accreted to form our planet in the early history of the solar system. Analyses of hydrogen isotopes in olivine-hosted melt inclusions and nominally anhydrous minerals point to a deep mantle reservoir that predates late accretion[25]. These findings suggest that at least part of Earth’s water was already present in the mantle rather than being solely imported. This interpretation is consistent with the oxygen isotope composition of primitive sodic TTGs derived from source rocks hydrated by primordial (mantle-derived) H2O[26]."
Line 72: The opening sentence is quite broad relative to the dataset presented. Consider clarifying that the study examines crust-forming processes through a case study of the Lewisian Gneiss Complex.
Lines 76-78: This sentence introduces an interpretation of the hornblendites. Consider clarifying whether this represents an observed field relationship or a petrogenetic interpretation discussed later.
Lines 101-107; Numerous numerical values are embedded directly in the text, which makes the section read like a table written as prose. Consider presenting the compositional data in tables and using the text to emphasise the main compositional characteristics and relationships.
Lines 109-110: Long lists of sample identifiers interrupt the flow of the Results section. Consider moving the full list of sample IDs to a table and referring to the dataset more generally in the text. Better to write: "The associated hornblendite pods commonly exhibit… (samples listed in Table X)"
Line 131: The zircon results contain valuable information but are difficult to follow because large amounts of numerical data and several analytical parameters are embedded directly in the text. Summarising zircon populations and key parameters in tables would significantly improve readability.
Line 169: The description of zircon populations (G1–G3 and G1’–G3’) is difficult to follow in the current prose format. A table summarising the populations, ages, number of analyses, and key geochemical characteristics would greatly improve clarity.
Line 196: Main faults in Discussion:
1. Repetition of the results instead of interpretation.
2. The argument is not clearly structured. A strong Discussion usually follows a logical structure:
Main findings
Interpretation of each key dataset
Comparison with previous studies
Implications for the broader problem
Limitations
3. Too much description of data plots.
4. Strong conclusions relative to the evidence. Several interpretations are presented as firm conclusions without clearly demonstrating how they are derived from the observations.
5. Lack of synthesis: The Discussion may contain many individual interpretations, but lacks a clear synthesis.
6. Overly long and dense sentences.
7. Results and discussion boundaries are blurred.
The key problem: The discussion is largely descriptive rather than analytical. Instead of answering what the results mean, it repeatedly describes what the results are.

Open Access This Peer Review File is licensed under a Creative Commons Attribution 4.0 International License, which permits use, sharing, adaptation, distribution and reproduction in any medium or format, as long as you give appropriate credit to the original author(s) and the source, provide a link to the Creative Commons license, and indicate if changes were made.
In cases where reviewers are anonymous, credit should be given to 'Anonymous Referee' and the source.
The images or other third party material in this Peer Review File are included in the article's Creative Commons license, unless indicated otherwise in a credit line to the material. If material is not included in the article's Creative Commons license and your intended use is not permitted by statutory regulation or exceeds the permitted use, you will need to obtain permission directly from the copyright holder.
To view a copy of this license, visit https://creativecommons.org/licenses/by/4.0/

Authors’ response to reviewers’ comments

Manuscript title: **Insights into early Earth continental crust formation from the most ancient heart of Scotland**

Silvia Volante, Fernanda Torres-Mancinelli, Jonas Kaempf, Vitor Barrote, Tim Johnson, Chris Kirkland, Maria R. Scicchitano, Lorenzo Tavazzani, Sampriti Basak, Anne-Sophie Bouvier, Annika Dziggel, Axel Gerdes

Manuscript number: NCOMMS-25-54974

Date: October 2025

Dear Editor and Reviewers,

Please find responses to review comments below (in blue font). Comments are reproduced in black font, quoting, where relevant, amended text (also in *blue*). Line numbers reflect the revised manuscript *without* tracked changes.

Sincerely,

Silvia Volante and co-authors

Reviewer–General comments:

Reviewer #1 (Remarks to the Author):
First, I would like to disclose that I have previously worked with some of the authors during my time at Curtin University several years ago. While this may be considered by some as a potential conflict of interest, I decided to take that and provide an objective and fair review.
The authors present new whole-rock geochemical data, along with zircon U-Pb, O, Hf, and trace element analyses, from two TTG samples (??) and seven hornblendite samples. The study reports the discovery of a ca. 3.7 Ga TTG–(ultra)mafic rock association, which represents a significant and exciting finding for the early Earth research community. Notably, the authors suggest that the hornblendite (ultra)mafic rocks may be relics of the early mafic crust.
While the analytical data presented are of high quality, I find the discussion on the origin and petrogenesis of both the TTG and hornblendite suites to be limited and in need of further elaboration. The TTG interpretations are particularly constrained by the small number of samples (only two), which warrants more careful contextualization.
Re: We appreciate the positive assessment and the suggestion to elaborate further on the samples petrogenesis.
In this revision we (i) expanded mineralogical descriptions for both TTG and hornblendite samples, and (ii) added per-analysis O-isotope and trace-element plots that underpin the age populations used in our discussion.
While our TTG dataset comprises two samples, they are incredibly important and have been investigated in considerable detail. The objective of this contribution therefore is to document a well-contextualized discovery from a focused area of the LGC, that has widely significant implications, and to extract process insights from this integrated petrography–geochemistry–geochronology. These discoveries provide key insights in the petrogenetic processes of felsic crust formation on the early Earth.

An intriguing observation is that the oldest zircon population in the hornblendites appears to be slightly older than (with high-resolution separation of the ages) Group 1 zircons in the TTG samples. This raises the possibility that the (ultra)mafic rocks may have served as a source for the TTGs, even those rocks found as xenoliths within TTG rocks, which highlights the assimilation later. I encourage the authors to explore this hypothesis further, potentially through detailed geochemical modelling and thermodynamic simulations.

Re: We thank the reviewer for this suggestion. Geochemical modelling and thermodynamic simulations are being addressed in a separate manuscript by the second author. This allows the present contribution to remain focused on petrological, geochemical and geochronological characterisation while acknowledging the potential of a source link as raised here.

Additional minor comments:

• Line 195 & 212: The authors mention "zircon growth in a water-rich environment." Please elaborate on this point and consider citing relevant literature to support the interpretation.

Re: We clarify the basis for inferring fluid-rich conditions (zircon trace-element systematics and textures consistent with hydrous magmatic to high-T fluid interaction) and point readers to the new trace element plots integrated in Fig. 3 and to CL/BSE images in the Supplementary Information. We have also added relevant literature as requested by the reviewer.

Lines 218–228:

Although ultramafic–mafic rocks are typically zircon-poor due to low Zr content, hornblendite pods in the LGC contain large zircon crystals (up to ~700 µm). These yield ages of c. 3690 Ma (G1'), c. 3470 Ma (G2'), and c. 2840 Ma (G3'). Zoned cores from the older grains evidence resorption textures by younger rims and the Th/U values decrease from 0.9–0.7 in the older grains to ~ 0.1 in the youngest c. 2840 Ma zircon population (G3'). Rims with $^{207}Pb/^{206}Pb$ dates of ca. 3470 Ma likely formed during the same event recorded by G2 hbl-TTG zircon population. The zoning, TE and size of the ca. 3690 Ma zircon grains (G1') indicate crystallization in a hot, water-rich environment[38] (Fig. 3), whereas the patchy zoning from rim to core of G2' may support a hydrothermal/metamorphic origin for this age population (see Supplementary Information, Supplementary Fig. 5). The younger cores and rims (c. 2840 Ma) align with the major crust-forming event in the LGC[28,39].

• Line 133: It would be helpful to include Eu* values in the discussion of geochemical characteristics.

Re: Eu* is now reported where REE patterns are discussed in the main text, with cross-references to the relevant tables/figures.

Lines 146–150:

Cathodoluminescence images indicate that eight analyses with 207Pb/206Pb dates ranging between 3485 ± 26 Ma and 3351 ± 27 Ma represent core–rim mixtures. Overall, G1 and G2 zircon grains/domains are slightly more enriched in HREE with G1 showing a more pronounced negative Eu anomalies (Eu=Eu/SQRT(Sm*Gd), with Eu*= 0.28–0.70) than Group 2 (Eu*= 0.04–0.4) and 3 (Eu*= 0.1–0.6; Fig. 3A).*

• Line 116: Consider comparing your hornblendite data with average compositions of both depleted and enriched Archean tholeiitic basalts.

Re: We added the average compositions of depleted and enriched Archean basalts in the trace element diagrams of Fig.1 as requested by the reviewers.

• Line 57: Please correct "meteoric" to "meteorite."

Re: Corrected.

Regards,
Hamed Gamaleldien

Reviewer #2 (Remarks to the Author):

Dear Authors

This is a surprising and exciting discovery. I was certainly not expecting TTGs and associated hornblendites of this age to crop out in the Lewisian and I think the dual ages of these components is certainly a big deal and worthy of publication in a widely read journal.

You have clearly given location lat and long for the samples in the appendices, which is good. I wonder if a simple geological map in the appendices could be added as I'm quite sure that many would like to see that too.

Re: Thank you for the constructive comments. We have added a simplified geological map of the study area together with a processed orthoimage of the investigated outcrops in the Supplementary Information and reference it from the field-related section of the main text.

I do think there could be a bit more clarity on the interpretation of the G1 and G2 ages from both TTG and hornblendite. It is not entirely clear whether you are invoking the former as being intrusion ages and the

latter as magmatic or metamorphic ages. Is the simplest explanation that the G1 ages are intrusive and the latter are metamorphic? Why are you being circumspect on this? I also wonder if you have tried some zircon trace elemental ratios other than Th/U to try and elucidate the source e.g. Hf vs U/Yb? This might also add to the debate on mantle vs crustal input.

Re: We have generated additional plots—U/Yb vs Hf, U/Yb vs Nb/Yb, Th/U vs $^{207}Pb/^{206}Pb$ Ages and Ti vs $^{207}Pb/^{206}Pb$ Ages—presented in Fig. 3 (main text) and Supplementary Information, Supplementary Fig. 5. While Th/U alone is not diagnostic in high temperature metamorphic zircon grains, most values exceed 0.1 (commonly used as a metamorphic threshold), and hydrothermal zircon commonly falls between magmatic and metamorphic fields. However, a systematic trend from G1 to G2 is observed in both TTGs and hornblendite. Integrated with CL/BSE textures, this implies:

- Importantly, the G2 population in TTG samples occurs mainly as oscillatory-zones zircon cores or as rims on c. 3600 Ma cores (Supplementary Information, Supplementary Fig. 3). These textures are characteristic of magmatic zircon. Although Th/U alone is not a robust discriminator, especially in high grade rocks, the G2 population averages ~0.5, within the typical magmatic range. We cannot exclude a high-T metamorphic origin for parts of the c. 3530 Ma G. Overall the weight of evidence favours magmatic growth.
- By contrast, CL and BSE textures for the G2' population (3.5 Ga) for the hornblendite samples show patchy zoning from rim to core or brighter rims, consistent with interaction of the magmatic zircon with fluids and a hydrothermal / high temperature metamorphic origin.

These observations are incorporated in the main text.

It now reads:

Results section (Lines 132-175):

"The hbl-TTG contains magmatic zircon cores up to c. 3700 Ma old, with 20 concordant (<5% discordance) $^{207}Pb/^{206}Pb$ dates older than 3300 Ma (see Supplementary Information, Supplementary Figs. 2 and 4). These core domains display oscillatory zoning typical of magmatic zircon, which are partially dissolved and characterised by overgrowths that also exhibit oscillatory zoning (Fig. 2A). Three zircon populations are identified based on internal textures, Th/U ratios, and relative TE content (Figs. 2C–D and 3A–E). Group 1 (G1) represents the oldest zircon population with five analyses that define a concordia age of 3632 ± 30 Ma (MSWD = 1.2, n = 5; all age uncertainties are presented at 95% confidence—2SE) and Th/U ratios between 0.62 and 0.76 (median = 0.7; Fig. 3B). Group 2 (G2) zircon comprises seven analyses which yield a concordia age of 3526 ± 15 Ma (MSWD = 0.76, n = 7) with Th/U ratios between 0.30 and 0.66 (median = 0.46; Fig. 3B). Group 2 analyses are either rims on c. 3600 Ma cores or concentrically zoned cores surrounded by c. 3480 Ma rims. Group 3 (G3) is the dominant population and defines an over-dispersed concordia age of 2779 ± 10 Ma (MSWD = 6.7, n = 73), where high MSWD indicates radiogenic-Pb remobilization. Also, recent Pb-loss events contributing to discordance towards c. 500 Ma are observed in Figure 2C. Cathodoluminescence images indicate that eight analyses with $^{207}Pb/^{206}Pb$ dates ranging between 3485 ± 26 Ma and 3351 ± 27 Ma represent core–rim mixtures. Overall, G1 and G2 zircon grains/domains are slightly more enriched in HREE with G1 showing a more pronounced negative Eu anomalies (Eu=Eu/SQRT(Sm*Gd), with Eu*= 0.28–0.70) than Group 2 (Eu*= 0.04–0.4) and 3 (Eu*= 0.1–0.6; Fig. 3A).*

Zircon grains within the hornblendite are sparse but are commonly found within the core domains of the hornblendite pods and display a diverse range of morphologies, including rounded (e.g., zrn1) and elongated prismatic crystals (e.g., zrn3) that are up to ~700 µm long and have complex internal zonation with discontinuous rims (Fig. 2B, and see Supplementary Information, Supplementary Fig. 5). Three zircon populations are identified based on internal textures, Th/U ratios, and relative TE content (Figs. 2E–F and 3A–E). Group 1' (G1') represents the oldest zircon population with eleven analyses that define a concordia age of 3692 ± 15 Ma (MSWD = 4.5, n = 19) and Th/U ratios between 0.5 and 1.0 (median = 0.8). Group 2' (G2') zircon comprises three analyses which yield a concordia age of 3466 ± 57 Ma (MSWD = 7.2, n = 4) with Th/U ratios between 0.23 and 0.5 (median = 0.4). Zircon rims at c. 3500 Ma (G2') are consistent with the G2 population for the hbl-TTGs. Only one $^{207}Pb/^{206}Pb$ date of 3419 ± 40 Ma is found for the outermost bright rim of a zircon grain with distinctively lower Th/U ratio of 0.09. Finally, Group 3' (G3') from CL-bright zircon cores defines a concordia age of 2835 ± 29 Ma (MSWD = 6, n = 9), with Th/U ratios between 0.1 and 0.7 (median = 0.1). The high MSWDs are consistent with over-dispersed data, which may indicate more than one episode of radiogenic-Pb loss. Concordant grains forming G1'–G3' populations exhibit distinct negative Eu anomalies (Fig. 3C).

In addition to REE patterns, geochemical discriminator plots of Hf versus U/Yb and Nb/Yb versus U/Yb and their covariation, provide further information about zircon crystallization environments and sources[30,31]. For both hbl-TTGs (G1, G2, and G3) and hornblendite (G1', G2', and G3') zircon

populations, a progressive enrichment in Hf and U, relative to Yb, promotes higher Hf concentration and an increase in the U/Yb ratio of the younger zircon grains (Fig. 3D). The Nb/Yb vs U/Yb diagram shows that, while G1' plots on the mantle-zircon array, G2' and G3' progressively distribute on a vertical trend in the field of 'magmatic arc-type (hydrous)'. For the hbl-TTGs, G1 falls within the field of 'magmatic arc-type (hydrous)', whereas both G2 and G3 fall towards the upper limit of the mantle array straddling towards the 'magmatic arc-type (hydrous)' field (Fig. 3E)."

Discussion section (Lines 202-228): *""*
"Here, we report zircon ages from hornblende-bearing high-MgO TTGs and enclosed hornblendite pods older than 3600 Ma, which provide the first direct evidence for the existence of evolved Eoarchaean crust in NW Scotland. The identification of a crustal component half-a-billion years older than previously recognised highlights the important role of high-MgO ultramafic–mafic rocks in the generation of primitive continental (TTG) crust and provides a temporal link between the LGC and the rest of the North Atlantic Craton. Zircon populations from the hbl-TTG yield ages of c. 3630 Ma (G1), c. 3520 Ma (G2), and c. 2800 Ma (G3). A positive correlation between G1 and G2 and Th/U ratios indicates that older grains generally have higher Th/U values. G3 reports a wide range in Th/U > 0.1 likely indicating hydrothermal and magmatic zircon growth during crust formation. Texturally, G1 and G2 zircons appear as oscillatory zoned cores partially overgrown by G3 rims, consistent with inherited relicts of eroded or buried crustal material and indicate the presence of nearby Eoarchaean crust[37]. Combined, these observations may further support the magmatic origin for G1 and G2, although a metamorphic origin for G2 cannot be discarded. Distinct εHf$_{(zircon)}$ evolution trends for G1–G2 versus G3, together with a juvenile component at c. 2800 Ma, indicate that the older zircon populations G1–G2 are best explained as inherited from nearby older crust, which was subsequently recycled by a younger magmatic episode (Fig. 4).
Although ultramafic–mafic rocks are typically zircon-poor due to low Zr content, hornblendite pods in the LGC contain large zircon crystals (up to ~700 µm). These yield ages of c. 3690 Ma (G1'), c. 3470 Ma (G2'), and c. 2840 Ma (G3'). Zoned cores from the older grains evidence resorption textures by younger rims and the Th/U values decrease from 0.9–0.7 in the older grains to ~ 0.1 in the youngest c. 2840 Ma zircon population (G3'). Rims with $^{207}Pb/^{206}Pb$ dates of ca. 3470 Ma likely formed during the same event recorded by G2 hbl-TTG zircon population. The zoning, TE and size of the ca. 3690 Ma zircon grains (G1') indicate crystallization in a hot, water-rich environment[38] (Fig. 3), whereas the patchy zoning from rim to core of G2' may support a hydrothermal/metamorphic origin for this age population (see Supplementary Information, Supplementary Fig. 5). The younger cores and rims (c. 2840 Ma) align with the major crust-forming event in the LGC[28,39]."

For the Hf isotopes, an obvious question where you have datasets with U-Pb ages that are spread and have some discordance is whether you have performed discordance tests against 176Hf/177Hf and EHf as that could account for some of the spread in data that you explain in a slightly complex way regarding sources and input of later juvenile additions vs much later reworking in the Neoarchaean. I would personally recommend including such a figure in the supplementary files along with a few words discounting (if that's provable) this as a mechanism for some of the spread in Hf isotopes in the G1 and G2 zircons.
Re: This was implemented. Two plots illustrating the initial Hf and εHf versus individual $^{207}Pb/^{206}Pb$ ages were included in the supplementary material (Supplementary Information, Supplementary Fig. 7). This figure illustrates that while minor Pb-loss is present across populations, the εHf spread requires mixing of different sources. This is especially for G3 at 2.8 Ga during crustal growth, where we infer an additional juvenile input. We added a concise note in the Discussion to separate analytical disturbance from source mixing.

The O isotope story is intriguing, but I think plotting all zircons rather than just median values might be useful. In doing this you have implied that the Neoarchaean zircons are more mantle like than the Eoarchaean ones from which surely you are reworking material? This would require Neoarachean mantle input which is juvenile but has Eoarchaean reworked crustal material in its source and therefore that the TTG actually has a Neoarchaean intrusive age? This does have a bearing on your interpretation of the older zircons as being from a wetter source so I think there should be internal consistency in your argument.
Re: We added per-analysis $\delta^{18}O$ plots by sample and $\delta^{18}O$ vs $^{207}Pb/^{206}Pb$ age (Supplementary Information, Supplementary Fig. 6). We clarify that Neoarchean zircons trending to mantle-like values imply juvenile input at ~2.8 Ga, whereas older inherited components show more variable $\delta^{18}O$; this is internally consistent with εHf interpretations.

I note also that you don't have any Hf for G3 hornblendite zircons which might have shed some light on the relationship between these two different lithologies and their evolution in Neoarchaean times. Is this because the rims were too small? I think this is worthy of mention.
Re: Suitable rims for Hf analysis were too small in the current mounts (and some grains were partly polished away between analytical sessions). We now state this limitation explicitly. We report one hornblendite zircon at ~2.8 Ga with $\delta^{18}O \approx 5.8‰$ as context.

In the supplementary files I think the data are overall transparent. I do think that some plots for the U-Pb RMs in S4 and S5 would be useful. Also, in S5, the ETH spreadsheet does quantify total external uncertainty at 1% for all RMs plus 0.5% long term excess, which are propagated in quadrature to all unknowns. I wonder where these figures come from as they are all the same and seem more like an estimate rather than being strictly quantified. Could you comment on this in the methods?
Re: Thanks for the positive assessment of our U-Pb data reporting. We have added some TW and WM plots for VRMs in S4 (see "U-Pb (VRMs)" sheet) and S5 (see new sheet "All_VRMs_excess_unc."). For S5 (U-Pb data from ETH Zürich), to provide the most accurate results, the (1) reproducibility of validation reference materials and (2) systematic long-term uncertainties are propagated to the weighted average uncertainties by quadratic addition (i.e., total external uncertainty). The results of the validating reference materials show the reproducibility of the method, which is in the range of 1 % (see new sheet "All_VRMs_excess_unc."). The long-term external uncertainty for zircon U-Pb analyses at the ETH LA-ICP-MS lab is in the range 0.5 % for $^{206}Pb/^{238}U$ ages and is composed of the uncertainty from the applied corrections, uncertainty of the decay constants, the uncertainty on the true $^{206}Pb/^{238}U$ ratio of the primary standard GJ-1, and possible uncertainty from matrix effects (Sliwinski et al., 2022). As the individual dates used to calculate final ages are $^{207}Pb/^{206}Pb$ dates rather than $^{206}Pb/^{238}U$, we consider the 0.5 % propagated uncertain a maximum estimate.
Ref: Sliwinski, J. T., Guillong, M., Horstwood, M. S. A. & Bachmann, O. Quantifying Long-Term Reproducibility of Zircon Reference Materials by U-Pb LA-ICP-MS Dating. Geostandard Geoanalytic Res 46, 401–409 (2022).

I also noted that in S6 in the FIERCE Frankfurt RMs worksheet that you have labelled the second set of zircons as GJ-1 (cell A48) but these have an assigned age of 417 Ma so either the age is wrong or you have mislabelled Temora as GJ-1?
Re: Thanks for spotting the mislabelled RM. The one used was Temora. We have corrected this in the revised Supplementary Information, Supplementary Fig. 6.

Overall, this is an exciting discovery and is supported by good data. The ms is written well with good figures and clear supplementary files. I hope my comments might help you to improve a bit further the paper with not much extra work.

Best wishes
Craig Storey

Reviewer #3 (Remarks to the Author):

The manuscript claims to provide evidence for the preservation of amphibolitized Hadean crust as enclaves in Eoarchean TTG gneiss. The mere discovery of an Eoarchean component in the Lewisian gniesses, supporting hypothetical associations with the North Atlantic Craton in the Archean, is an important discovery on a local scale. However, the paper is more ambitious than that, and seeks to establish that preserved Hadean mafic protocrust is preserved, in similar fashion to the contested claims of Hadean faux-amphibolite in far northeastern Canada have done. However the evidence in comparison is limited to the interpretation of small pods of amphibolite embedded in felsic gneiss, unlike the substantial tracts of mafic rocks found at Nuvvuagittuq.

Firstly, it is just misleading to claim that the hornblendites "represent vestiges of a primitive hydrated (proto)crust", when the oldest zircon age estimate is only 3.7Ga and model ages along a 'default' depleted mantle array range from 4.1 to 3.9Ga. Without careful reading, this gives the impression that the rocks themselves are Hadean, which the authors know is not true. Beyond the assumption that such an assumed

mantle array requires continual crustal growth from the beginning of the Earth that approximates in composition modern continental crust, an idea whose time has long since passed, a vague estimate for mantle derivation around 4Ga is hardly evidence that the crustal source of melting is older than 4Ga, let alone the age of the amphibolite itself.

Re: We appreciate this comment and concur that model ages do not represent crystallization ages. We have clarified the wording to avoid any potential miss reading that the hornblendites themselves are Hadean.

We state explicitly that the measured zircon populations in hbl-TTG and hornblendite are Eoarchean (c. 3.7-3.6 Ga) and that any Hadean source involvement is inferred only from model ages integrated with petrography and geochemistry—not from direct rock ages. The discussion emphasizes the interpretive limits of model ages while explaining how they inform our preferred petrogenetic scenario. We note that this approach is the community standard for inferring source components and is supported by oxygen isotope data that provide a mechanism to understand the involvement of source mixtures.

A basic problem in the chain of logic can be found simply by looking at the photographic evidence – these enclaves are not simply amphibolites in the strict IUGS definition, but coarse-grained mesocratic mafic pods that are not properly described in sample description section – here the authors merely mention the presence of amphibole, whereas the field and microphotos show pods with an abundance of felsic minerals along with amphibole, which they vaguely describe as 'pegmatitic' without explanation.

Re: Thank you for giving us the opportunity to address this potential ambiguity. We consistently describe the pods as hornblendite (>95 vol% hornblende) rather than amphibolite—which would require plagioclase in comparable abundance). This is documented in Fig. 2A and in the Supplementary Information, Supplementary Fig. 3).

We now replace the term "pegmatitic" with a descriptive phrase—"prismatic hornblende crystals up to 5 cm"—to avoid any petrogenetic implication. The revised paragraph (quoted in our response) specifies that the pods are essentially monomineralic hornblendite with minor interstitial phases (quartz, chromite, Fe–Ti oxides, zircon).

The current text reads as follow (Lines 86–132):

In-situ U–Pb, TE, Lu–Hf and O isotope analyses were performed on zircon from hornblende-bearing tonalite gneisses (hereafter hbl-TTG) and enclosed hornblendite pods (Fig. 1A–C) from the Achnasheen–Gruinard locality in the Gruinard terrane of the LGC[28] (see Supplementary Information, Supplementary Figs. 1 and 2). At outcrop scale, the hbl-TTG are weakly deformed, coarse-grained, and composed mostly of plagioclase, quartz and hornblende (Fig. 1A), contrasting with the typical, strongly deformed and finer-grained banded grey gneisses that dominate the LGC[29]. The hornblendite pods are dark green, medium- to coarse-grained rocks and, essentially, monomineralic, dominated by granoblastic hornblende (≥95 vol.% hornblende), with minor accessory or interstitial phases including quartz, Cr–Ti–Fe oxides and zircon. These pods are weakly deformed to undeformed, up to two-metres across and have sharp contacts with the host hbl-TTG gneisses (Fig. 1A). Some hornblendite pods contain cores with prismatic hornblende crystals up to 5 cm long that transition into finer-grained rims up to a centimetre thick (Fig. 1B–C).

The hbl-TTG samples contain plagioclase, quartz, and hornblende with accessory zircon. Secondary biotite replaces hornblende, and apatite and rutile occur within biotite and amphibole–quartz intergrowths that replace hornblende or clinopyroxene (see Supplementary Information, Supplementary Fig. 3). The hbl-TTGs (samples LG2022 and LG2023B) have ~64 wt% SiO_2 and are sodic (K_2O/Na_2O < 0.4), with high Sr/Y ratios (>30), Mg# (49 and 67, where Mg# = 100 x atomic Mg/(Mg + Fe)), MgO (~3.0 wt%), Ni (44 and 168 µg/g) and Cr (48 and 214 µg/g) contents (Fig. 1D–F) relative to most TTGs[26,29]. Although these samples approach sanukitoid-like chemistry, a suite typically characterised by SiO_2 ≤ 60 wt.%, MgO > 6 wt.%, Mg# > 0.60, Cr > 100 µg/g, and Sr and Ba > 500 µg/g, our samples have slightly higher SiO_2 (65.15 and 63.23 wt.%), and lower MgO (2.23 and 3.26 wt.%). In primitive-mantle normalised trace element plots, the hbl-TTGs show pronounced negative anomalies for Nb, Ta and Ti, and positive anomalies for Pb, Zr, Eu and Sr (Fig. 1F).

The associated hornblendite pods (LG2023A, LG2023C, LGC2024-1a, LGC2024-1b, LGC2024-2, LGC2024-4a, LGC2024-4b) commonly exhibit large (~4 mm) granoblastic amphibole crystals with diffuse to patchy zoning, mantled by sub-mm-thick pale green acicular to prismatic actinolite rims. Fine-grained amphibole–quartz intergrowths mimic domains that may represent pseudomorphs after primary magmatic clinopyroxene/hornblende, but no relics were observed. When a core–rim structures at the pod-scale is present, cores contain randomly oriented, prismatic amphibole crystals that are partly replaced by acicular actinolite aggregates. Towards the rims, grain size decreases. Quartz, when present, occurs as thin films along amphibole grain boundaries or as small veinlets. Cr–Ti–Fe oxides are widespread, most enclosed in amphibole cores but also associated with actinolite rims. Rare subhedral zircon grains up to ~400 µm in size are enclosed in large amphibole crystals. These pods are characterised by 49–53 wt% SiO_2, 16–19 wt% MgO, 6–12 wt% CaO, and low TiO_2 (<0.2 wt%) with Mg# of 68–75. Hornblendite pods with texturally distinct core and rim structures (Fig. 1B–C) have FeO contents decreasing from core (13–16 wt%) to rim (11 wt%), increasing CaO (from 6–9 wt% to 11 wt%), and decreasing Ni contents (from >2000 to 500 µg/g). They exhibit Cr contents up to 5620 µg/g, Ni up to 2850 µg/g, and V between 20 and 105 µg/g. Elemental maps reveal Cr and Ti zoning in amphibole crystals with concentrations decreasing from core to rim (see Supplementary Information, Supplementary Fig. 3). These elements are also concentrated in Cr–Ti–Fe oxides (chromite), which occur as inclusions in amphibole cores. Primitive mantle-normalised TE patterns for the whole rock composition of the hornblendites reveal depletion in Cs, Rb, U, Ti, La, and Sr, mild enrichment in Ba, and flat middle to heavy rare earth element (M- to H-REE) patterns, with positive anomalies in Ba, Pb, Nd, Sm and Eu (Fig. 1G).

These pods have reaction mantles with the host TTGs that are almost monominerallic (hornblende with minor biotite and very little plagioclase or quartz), a strong indication that the assemblage is a product of metasomatic interaction with the granitoid host. This is supported by the chemical maps provided, which demonstrate mineralogical and chemical banding absolutely characteristic of reaction textures during metamorphism.

That mny pods are entirely hornblendite merly indicates that the original encvlave has been consumed during metamorphism. As such, interpreting these as some kind of 'primordial' amphibolite is highly suspect. Instead, the textures are entirely consistent with metamorphic mineral growth from basaltic, doleritic or even gabbroic xenoliths in a granitoid, with hydration accompanying metamorphism. If the zircon have only been found in the hornblendite rinds, and not in the 'pegmatitic' cores, it is possible that they all derive from the TTG host. The in-situ hosts of grains are nonetheless mafic, and the well-documented presence of zircon within them is important and needs explanation.

Re: We agree that hornblendite–tonalite reaction rims may form by interaction with hydrous magma. This does not preclude enclave cores being residual after fluid-fluxed melting of a basaltic–gabbroic source that produced tonalitic melts and left a water-rich residue later incorporated by the magmas; subsequent magma–enclave interaction plausibly generated the reaction rims.

Indeed, the monomineralic composition of the hornblendite does not indicate 'merely consumption of the original enclave by metamorphism'. Instead, this indicates extremely wet-conditions and constant interaction with fluids fluxing the mafic crust, breaking down all the felsic mineral and leaving behind a water-rich hornblende-bearing residue.

The investigated zircon grains are rare but sparse in the hornblendite samples, occurring within granoblastic hornblende, within symplectitic quartz-amphibole domains, and with retrograde biotite (these textural relationships can be observed in Fig. 2B as well as Supplementary Information, Supplementary Figs. 3 and 5). Most zircon grains are located in hornblendite pod cores. These relations support in-situ mafic association rather than derivation from the host. We added this to the text and figure descriptions.

The coarse, complex character of the grains fits with an association with a gabbroic or granitoid protolith, rather than just growth with the monominerallic hornblendite during metamorphism. The diversity in age texture and composition is attributed by the authors to multistage modification of originally magmatic zircon of ca. 3.7 age. However, not all the variability is easily relegated to this cause.

Re: We agree with the reviewer on the zircon grains showing complicated textures and chemical-age variations. Following Reviwer#2 we have added various trace element plots including Th/U vs 7/6 Age to further discuss the origin of these different populations.

Indeed, although the oldest population of 3.7 Ga (G1') is robustly supported not only by trace elements and trace element ratios but also by CL and BSE texture images, the population G2' of c. 3.5 Ga age has a more complicated record. Th/U ratios show slightly lower values for this population compared to G1'but still higher than 0.1, whereas other discrimination diagrams (such as U/Yb vs Hf) imply wetter conditions for this population. These lines of evidence support interpretations from BSE and CL images, which show patchy zoning from the rim towards the core, commonly associated to zoning formed by interaction with fluids.

It is claimed that both internal zircon textures and Th/U are used to discriminate between generations of zircon growth. There is no evidence presented in either figure or text that this is the case – we are left to look at the handful of grain images provided and work out how they were interpreted ourselves.

The documentation of relations between analyses and spot positions is better for the TTG but is obscured for the critical amphibolite data.

Re: We expanded CL/BSE image panels (Supplementary Information, Supplementary Fig. 4 and 5) and added Th/U vs $^{207}Pb/^{206}Pb$ age in Fig. 3B, linking spot positions, textures, and ages. Together, this documentation makes it explicit where textures and Th/U inform the assignment of zircon populations in TTG vs hornblendite.

All Hf data come from a single grain, which yielded variable Pb-Pb ages. The assumption made that age variation is a product of disturbance during metamorphism – and hence all Hf isotopic values should be calculated for the oldest age 'population' – can be tested by the variation in eHf that results: low variability would confer validity to the assumption. Instead, eHf scatters above and below chondritic values, invalidating the assumption. Isotopically, the diversity in eHf measurements at 3.7Ga is a red flag- it points to either multiple, poorly mixed sources of Hf or the erroneous assignment of all data to a single Eoarchean age. It certainly cannot be attributed to a single, so called "Hadean" depleted mantle source, nor could the higher values be derived from anything older than the late Eoarchean. The fact that all analyses come from a single grain even calls into question whether the zircon is magmatic or contains a xenocrystic component not associated with the host protolith.

Re: Hf was analyzed on several grains in both lithologies; we used the inferred age populations (G1/G2/G3; G1'/G2'/G3') to calculate initial εHf. Following Reviewer #2's advice, SI plots of εHf and initial Hf versus single-spot $^{207}Pb/^{206}Pb$ ages show that minor Pb-loss alone cannot explain the full spread, which requires mixed sources—especially for G3 (~2.8 Ga). We added text distinguishing analytical disturbance from geological mixing.

The slightly heavier oxygen isotope values from the amphibolite may seem meaningful if the dta from this study is taken by itself, but is well within the variation commonly seen in Eoarchean magmatic protoliths in the literature. As such there is no significant meaning in the variation seen.

Re: As also recommended by Rev#2 we added plots that shows each individual analysis for O-isotope for each investigated sample (see Supplementary Information, Supplementary Fig. 6). We agree that the variability is not statistically different between the individual O-isotope analysis of the investigated hbl-TTG and hornblendite and we do not claim a turnaround of the story because of different O-isotope between the identified population. In contrast, what this data shows is how the felsic crust formed in constant presence of mantle-derived fluids. We emphasize that most zircons are mantle-like to slightly elevated, consistent with dominantly mantle-derived fluids plus subordinate fractionated inputs, consistent with global early Earth TTG compilations.

There is important work here, carefully documented. It has importance for the relationship between Scotland and other Archean components of the North Atlantic Craton. However the authors have chosen to use good data stretched thinly over a frame thin interpretation. A more humble approach would make this a great paper.

We thank Reviewer 3 for recognising the importance of the dataset and its relevance to the relationships between Scotland and other Archaean components of the North Atlantic Craton. We fully agree that interpretations should not outrun the resolving power of the evidence, and we have revised the manuscript accordingly to ensure that our conclusions are more clearly and explicitly bounded by the data. Yet we consider it important to frame the work within a process orientated framework that is widely relevant, not to do this underutilises the data.

In the revised version, we (i) temper some of the broader tectonic statements and frame them as hypotheses rather than firm conclusions, (ii) make the key inferences more explicitly traceable to specific observations (e.g. zircon age–Hf arrays, isotopic comparisons and structural relationships), and (iii) expand the discussion of alternative explanations and remaining uncertainties. These changes are intended to remove any impression that "good data [are] stretched thinly over a frame-thin interpretation" and instead emphasise a conservative reading of the results.

With these modifications, we believe that the carefully documented dataset now supports the principal interpretations in a more transparent and proportionate way. The paper retains its core message on the affinities between Scottish crust and other elements of the North Atlantic Craton but presents this in a more measured tone that we hope addresses Reviewer 3's concern about over-extension.

Other comments

On line 140-1 there isa claim to support interpretation with texture and Th/U – yet there is nothing in the diagrams referred to support such a claim.

Re: We add a Th/U vs Ages binary plot in Fig. 3 in the main text.

The Group one pooled age for LGC2023A is misleading – an MSWD of almost 5 for 18 data is a clear failure of the test of equivalence. This is partly due to the inclusion of one older outlier - the remaining data are to the age derived from the host TTG. Thus the interpretation of an older amphibolite in a pre-metamorphic TTG host rock is biased by one data point. It does not eliminate the possibility that zircon in the mafic enclave is actually from the TTG host.

Re: We apologize for the earlier figure omission. The calculation for the older population was correct, but we realised that the plot in Fig. 2E did not represent all the analysis obtained for hornblendite samples. re-plotted concordia for all hornblendite populations. G1' (n=19) now spans 3650–3750 Ma, with several analyses > 3700 Ma—ages not observed in the hbl-TTG zircon. The added initial Hf vs 7/6 individual ages plot (Supplementary Information, Supplementary Fig. 7) clearly shows that ancient Pb-loss (commonly documented in the LGC and other high-T terranes; Whitehouse et al., 2010; 2022; Tedeschi et al., 2023; Taylor et al., 2020) likely contributes to dispersion.

The slightly heavier oxygen isotope values from the amphibolite may seem meaningful if the dta from this study is taken by itself, but is well within the variation commonly seen in Eoarchean magmatic protoliths in the literature. As such there is no significant meaning in the variation seen. For oxygen isotope values near the upper mantle limit ($\delta^{18}O$ of ca. 5.9 ‰) the authors invoke interaction between "surface water" and magma, but do not specify the isotopic composition of such water (is it SMOW?). In fact, surface-derived water commonly has sub-mantle values ($\delta^{18}O$ < 4.9 ‰) and meteoric water has $\delta^{18}O$ as low as –30 ‰. High-temperature interaction with low- $\delta^{18}O$ waters would lower primary, mantle-like $\delta^{18}O$ rather than elevate it. By contrast, crustal materials that interacted with surface water, for example sediments, acquire supra-mantle $\delta^{18}O$. An easier explanation for the elevated zircon $\delta^{18}O$ is assimilation of such sediments into the magma.

Re: We agree that zircon $\delta^{18}O$ near the upper-mantle limit does not by itself imply Hadean crustal input. We specify that surface-derived waters can be low-$\delta^{18}O$, and high-T interaction would lower mantle-like $\delta^{18}O$. Elevated zircon $\delta^{18}O$ is more parsimoniously explained by assimilation of crustal materials that had low-T exchange with the hydrosphere; we now state this explicitly, with references (Eiler, Valley, Bindeman). We also clarify that our main conclusion is consistent with zircon growth in equilibrium with dominantly mantle-derived fluids during its growth plus subordinate fractionated inputs. This evidence highlights that zircon grew in a wet environment in equilibrium with mantle-derived fluids.

The authors do not clearly state in the manuscript the true age of these TTG rocks nor whether the ca. 3.64 and ca. 3.52 Ga zircon populations are inherited/xenocrysts or represent the protolith crystallization age. The ages of ca. 2.8 Ga were also obtained from oscillatory-zoned domains which include older cores suggesting inheritance. In the Fig. 2C, a substantial number of analyses plot discordantly towards ca. 500 Ma on the concordia diagram. This should be also commented on in the text.

Re: We clarified in the Discussion that TTG zircons define populations at c. 3630 Ma (G1), c. 3520 Ma (G2), and c. 2800 Ma (G3). G1–G2 typically occur as oscillatory-zoned cores overgrown by G3 rims, consistent with inheritance from nearby Eoarchean crust; combined textures and Th/U support a magmatic origin for G1–G2, while a high-T metamorphic origin for parts of G2 cannot be excluded. We also note and briefly discuss analyses that plot discordantly toward c. 500 Ma in Fig. 2C.

Lines 208–218:

"Zircon populations from the hbl-TTG yield ages of c. 3630 Ma (G1), c. 3520 Ma (G2), and c. 2800 Ma (G3). A positive correlation between G1 and G2 and Th/U ratios indicates that older grains generally have higher Th/U values. G3 reports a wide range in Th/U > 0.1 likely indicating hydrothermal and magmatic zircon growth during crust formation. Texturally, G1 and G2 zircons appear as oscillatory zoned cores partially overgrown by G3 rims, consistent with inherited relicts of eroded or buried crustal material and indicate the presence of nearby Eoarchaean crust37. Combined, these observations may further support the magmatic origin for G1 and G2, although a metamorphic origin for G2 cannot be discarded. Distinct εHf(zircon) evolution trends for G1–G2 versus G3, together with a juvenile component at c. 2800 Ma, indicate that the older zircon populations G1–G2 are best explained as inherited from nearby older crust, which was subsequently recycled by a younger magmatic episode (Fig. 4)."

In-line comments:

L30: add the information about the age of TTG. Also, the two felsic samples are tonalitic, not "TTG"

Re: We have added the age populations for both tonalite and hornblendite and modified hbl-TTG to hbl-tonalite.

L35-36: δ18O of 6.3 ‰ does not require contribution of Hadean continental crust — so it's better to move this information after εHf. Also elevated δ18O requires interaction with crustal material itself rather than surface-derived water.

Re: Edited accordingly.

L92: Here the authors state that hbl-TTG contains plagioclase, quartz and hornblende, while in L98 state that it contains hornblende, plagioclase and quartz. Then, which is the most common mineral in these two hbl-TTGs?

Re: Standardized to "plagioclase, quartz, hornblende."

L104-105: In Figs. 1D,E the studied "TTG" are more similar to sanukitoids. Can authours add an comment on that?

Re: We added a sentence reporting similarities and differences relative to sanukitoid-like chemistry.

L187: How the presence of a juvenile component support the interpretation of G1 and G2 as magmatic relics?

Re: We clarify that εHf trends are not used to demonstrate magmatic origin for G1–G2—that inference is supported independently by zircon textures and chemistry. Instead, the distinct εHf evolution of G1–G2 relative to G3, together with juvenile input at ~2.8 Ga, constrains provenance: the older zircon populations likely derive from nearby older crust recycled into younger magma.

L273-274: The authors state "interaction between surface water and magmatic processes". Surface water can interact with magmas or during magmatic processes.

Re: Edited accordingly.

L277: The term "low-temperature oxygen" is a shortcut and should be omitted. Please use "exchanged oxygen isotopes with hydrosphere at low temperatures".

Re: Edited accordingly.

Fig. 2F: "No residual plagioclase" should point to Sr, not Nd in the figure.

Re: Corrected.

Fig. 3B: it should spell "(Ce/Yb)N" on the y axis. This figure is not informative as the values of (Ce/Yb)N of the three zircon groups are similar. I propose to use age vs. Th/U – which is also commented in the text.

Re: We have changed this figure with the recommended plot of Th/U vs 7/6 individual ages in the main text.

Fig. S2: Does really the maps show weight percent? Not area percent?

Re: In nearly all case, information in the mining industry is discussed and calculated based on Mass % (wt%) and not area %. That is because the value of any given product, i.e. Cu, Au, etc etc is based on its mass and not its area. Therefore, area% only becomes useful or relevant, under certain circumstances. One example is with regards to porosity / permeability, for example in the oil industry, where they are interested in the porosity of the sample. This can only be measured or compared via area%. Other similar examples would be vesicles in volcanic rocks.
Therefore, I can confirm that we have checked all densities correctly and generated all mixes properly. Consequently, all maps show wt%.

Reviewer #4 (Remarks to the Author):

I co-reviewed this manuscript with one of the reviewers who provided the listed reports. This is part of the Nature Communications initiative to facilitate training in peer review and to provide appropriate recognition for Early Career Researchers who co-review manuscripts.

Re: We thank Reviewer #4 for co-reviewing. All points overlapping with Reviewer #3 have been addressed as detailed above.

Authors' response to reviewers' comments

Manuscript title: **Insights into early continental crust formation from the most ancient heart of Scotland**

Silvia Volante, Fernanda Torres-Mancinelli, Jonas Kaempf, Vitor Barrote, Tim Johnson, Christopher Kirkland, Maria R. Scicchitano, Lorenzo Tavazzani, Sampriti Basak, Anne-Sophie Bouvier, Annika Dziggel, Axel Gerdes

Manuscript number: NCOMMS-25-54974A

Date: March 2025

Dear Editor and Reviewers,

Please find responses to review comments below (in blue font). Comments are reproduced in black font, quoting, where relevant, amended text (also in *blue*). Line numbers reflect the revised manuscript *without* tracked changes.

Sincerely,

Silvia Volante and co-authors

Reviewer–General comments:

Reviewer #5 (Remarks to the Author):

Reviewer #5 (Remarks to the Author):

This manuscript presents new geochemical and isotopic data from hornblende-bearing TTGs and associated hornblendite pods from the Lewisian Gneiss Complex, and reports zircon populations older than 3.6 Ga. These data potentially address an important question concerning the formation of early continental crust and the preservation of ancient crustal components within the North Atlantic Craton. The study combines petrographic observations, whole-rock geochemistry, and in situ zircon geochronology and isotopic analyses, which together may provide useful constraints on early crustal processes.

However, despite the revisions made in response to previous reviews, the manuscript still suffers from substantial problems in structure, clarity, and scientific presentation. In its current form, the text is often difficult to follow and does not consistently adhere to common practices of scientific writing. These issues make it challenging to evaluate the scientific interpretations presented in the paper.

We thank the reviewer for the detailed assessment of the manuscript and for the suggestions regarding structure and clarity. Several of the comments relate to preferences in writing style and presentation rather than to issues affecting the interpretation of the data. We carefully evaluated these suggestions during revision. While we implemented changes where they improved clarity (see specific responses below), we maintained the current structure and presentation in several cases because we believe it already provides a concise and clear presentation of the dataset and interpretations, consistent with the journal guidelines and the absence of similar concerns from other reviewers.

The main concerns are summarised below.

1. Structure and organisation

The manuscript would benefit from a clearer separation between description, results, and interpretation. In several sections, particularly the Results and Discussion, descriptive observations, numerical data, and interpretation are interwoven. As a result, it is not always clear which statements represent observations and which represent interpretation, nor which interpretations are proposed by the authors and which are derived from previous studies. In addition, parts of the Discussion repeat descriptive information already presented in the Results rather than focusing on interpretation and synthesis.

Re: Some observations are briefly reiterated in the Discussion to guide the reader through the interpretation and to link specific datasets directly with the proposed interpretations. We believe this helps maintain the logical flow between observations and interpretation while keeping the Results section focused on reporting the analytical data.

2. Presentation of results
The Results section contains long passages listing numerical values, ratios, and sample identifiers directly in the text. This substantially reduces readability. Detailed numerical data and sample identifiers would be more effectively presented in tables, while the text should focus on highlighting patterns, contrasts, and relationships within the dataset.
Re: We appreciate the suggestion. Because the dataset is relatively small and the manuscript format is concise, we chose to present key numerical values directly in the text so that the reader can immediately see the analytical results without repeatedly consulting (supplementary) tables. The sentences in this section were kept short and focused on the main analytical parameters to maintain readability.

3. Clarity of writing
Many sentences are long and syntactically complex, which obscures the main message of the text. Simplifying sentence structure and breaking complex statements into shorter sentences would significantly improve readability and make the scientific argument easier to follow.
Re: We carefully reviewed the manuscript to identify sentences that might be overly long or syntactically complex. Several sentences were simplified or split into shorter statements to improve readability where appropriate.

4. Discussion
The Discussion remains largely descriptive and does not consistently develop a clear line of reasoning from the data to the proposed interpretations. Results are sometimes restated rather than interpreted, and interpretative statements are occasionally introduced without clearly explaining how they follow from the observations. A more focused Discussion that explicitly links observations, interpretations, and broader implications would strengthen the manuscript.
Re: We carefully re-examined the Discussion section considering this comment. Our intention is to summarise key observations and then link them to the proposed interpretations. While some observational elements are reiterated to maintain continuity with the Results, we clarified several passages to better highlight the interpretative aspects and the broader implications of the dataset.

5. Framing of the research question
While the Introduction provides useful background, the specific research question and objectives of the study could be articulated more clearly. A clearer formulation of the aims would help guide the reader through the Results and Discussion.
Re: We revised the final paragraph of the Introduction to more clearly state the objectives of the study and how the dataset from the Lewisian Gneiss Complex is used to address questions related to early continental crust formation.

Summary
The dataset presented in this manuscript may provide valuable insights into early crust formation in the Lewisian Gneiss Complex. However, substantial revision is required to improve clarity, structure, and adherence to standard practices of scientific writing. In particular, the separation between Results and Discussion should be clarified, detailed numerical data should be moved to tables where appropriate, and the overall narrative should be streamlined to emphasise the key observations and their implications.
Addressing these issues would greatly improve the readability of the manuscript and allow a clearer assessment of the scientific significance of the work.
Re: Most of the comments provided by this review are relate to the reviewers writing style preference rather than specific scientific points. We disagree over writing style. The other reviewers did not share similar issues about the readability of the text. Additionally, we believe that the current style not only reflects the journal guidelines, but it also clearly presents the results and interpretations in a coherent succinct form.

Detailed comments

Lines 36-38: Question - What are the structural relationships between the TTGs and the preserved vestiges in the field?

Re: We address field structural relationships between the TTGs and the hornblendite pods in the Sample description section due to space availability. The hornblendite are pods within the felsic TTG gneisses that are wrapped by the younger gneissic foliation. The latter developed during metamorphic overprinting events, and it is extremely weak in the studied area relative to other regions of the LGC.

Lines 42-43: The TTGs record not only deformation and metamorphism but also preserve primary structures related to partial melting.

Re: The phrase “variably deformed” includes low-strain domains where such primary textures related to partial melting may be preserved. We have clarified this point in the revised manuscript.

Lines 63-70: The original formulation is slightly confusing because both clauses refer to extraterrestrial bodies. The paragaph could be rephrased for clarity, for example: "The origin of Earth’s hydrosphere has long been explained by the delivery of water by comets and asteroids. However, a growing body of evidence suggests that much of the water may have been present earlier, sequestered within the numerous bodies that accreted to form our planet in the early history of the solar system. Analyses of hydrogen isotopes in olivine-hosted melt inclusions and nominally anhydrous minerals point to a deep mantle reservoir that predates late accretion[25]. These findings suggest that at least part of Earth’s water was already present in the mantle rather than being solely imported. This interpretation is consistent with the oxygen isotope composition of primitive sodic TTGs derived from source rocks hydrated by primordial (mantlederived) H2O[26]."

Re: We appreciate the suggested rewording, and we have edited the text accordingly.

Line 72: The opening sentence is quite broad relative to the dataset presented. Consider clarifying that the study examines crust-forming processes through a case study of the Lewisian Gneiss Complex.

Re: edited as suggested.

Lines 76-78: This sentence introduces an interpretation of the hornblendites. Consider clarifying whether this represents an observed field relationship or a petrogenetic interpretation discussed later.

Re: This sentence clearly reflects one of the main findings of this study. As per journal style the main findings are briefly summarized at the end of the introduction.

Lines 101-107; Numerous numerical values are embedded directly in the text, which makes the section read like a table written as prose. Consider presenting the compositional data in tables and using the text to emphasise the main compositional characteristics and relationships.

Re: We acknowledge this. However, because the dataset is limited in size, we present the key analytical values directly in the text to allow the reader to follow the results without repeatedly consulting tables. We believe this format keeps the Results section concise while clearly reporting the analytical data.

Lines 109-110: Long lists of sample identifiers interrupt the flow of the Results section. Consider moving the full list of sample IDs to a table and referring to the dataset more generally in the text. Better to write: "The associated hornblendite pods commonly exhibit... (samples listed in Table X)"

Re: The table where the samples are listed was cited here replacing the long list of samples.

Line 131: The zircon results contain valuable information but are difficult to follow because large amounts of numerical data and several analytical parameters are embedded directly in the text. Summarising zircon populations and key parameters in tables would significantly improve readability.

Re: We appreciate the suggestion. However, the main zircon populations and age distributions are already summarised in Figure 2 (particularly Fig. 2C and 2E), which provides a clear visual overview of the data. For this reason, we retained the current format in the text while referring readers to the figure for a summary representation.

Line 169: The description of zircon populations (G1–G3 and G1’–G3’) is difficult to follow in the current prose format. A table summarising the populations, ages, number of analyses, and key geochemical characteristics would greatly improve clarity.

Re: A summary of the zircon age populations is offered in Figure 2, which provides a clear visual overview of the data. For this reason, we retained the current format in the text while referring readers to the figure for a summary representation.

Line 196: Main faults in Discussion:
1. Repetition of the results instead of interpretation.
2. The argument is not clearly structured. A strong Discussion usually follows a logical structure:
Main findings
Interpretation of each key dataset
Comparison with previous studies
Implications for the broader problem
Limitations
3. Too much description of data plots.
4. Strong conclusions relative to the evidence. Several interpretations are presented as firm conclusions without clearly demonstrating how they are derived from theobservations.
5. Lack of synthesis: The Discussion may contain many individual interpretations, but lacks a clear synthesis.
6. Overly long and dense sentences.
7. Results and discussion boundaries are blurred.
The key problem: The discussion is largely descriptive rather than analytical. Instead of answering what the results mean, it repeatedly describes what the results are.
Re: We have again carefully evaluated these points during revision. Our intention in the Discussion is to combine key observations with interpretation to guide the reader through the reasoning that links the analytical results to the proposed petrogenetic model. We have clarified several passages to better emphasise interpretation and synthesis while retaining a concise structure appropriate for the manuscript length. We note that the manuscript was also evaluated by other reviewers who did not raise similar concerns regarding structure or clarity.
